# Blockage of Nrf2 and autophagy by L-selenocystine induces selective death in Nrf2-addicted colorectal cancer cells through p62-Keap-1-Nrf2 axis

**DOI:** 10.1038/s41419-022-05512-2

**Published:** 2022-12-20

**Authors:** Wei-Lun Hsu, Chieh-Min Wang, Chao-Ling Yao, Ssu-Ching Chen, Chung-Yi Nien, Yang-Ho Sun, Tsung-Yu Tseng, Yueh-Hsia Luo

**Affiliations:** 1 Department of Life Sciences, National Central University, Taoyuan, Taiwan; 2 Department of Chemical Engineering, National Cheng Kung University, Tainan, Taiwan

**Keywords:** Stress signalling, Targeted therapies

## Abstract

Persistent Nrf2 activation is typically noted in many cancers, including colorectal cancer (CRC), aiding cancer cells in overcoming growth stress and promoting cancer progression. Sustained Nrf2 activation, which is beneficial for cancer cells, is called “Nrf2 addiction”; it is closely associated with malignancy and poor prognosis in patients with cancer. However, Nrf2 inhibitors may have adverse effects on normal cells. Here, we found that the selenocompound l-selenocystine (SeC) is selectively cytotoxic in the Nrf2-addicted CRC cell line WiDr cells, but not in non–Nrf2-addicted mesenchymal stem cells (MSCs) and normal human colon cells. Another CRC cell line, C_2_BBe_1_, which harbored lower levels of Nrf2 and its downstream proteins were less sensitive to SeC, compared with the WiDr cells. We further demonstrated that SeC inhibited Nrf2 and autophagy activation in the CRC cells. Antioxidant GSH pretreatment partially rescued the CRC cells from SeC-induced cytotoxicity and Nrf2 and autophagy pathway inhibition. By contrast, SeC activated Nrf2 and autophagy pathway in non–Nrf2-addicted MSCs. Transfecting WiDr cells with *Nrf2*-targeting siRNA decreased persistent Nrf2 activation and alleviated SeC cytotoxicity. In *KEAP1*-knockdown C_2_BBe_1_ cells, Nrf2 pathway activation increased SeC sensitivity and cytotoxicity. In conclusion, SeC selectively attacks cancer cells with constitutively activated Nrf2 by reducing Nrf2 and autophagy pathway protein expression through the P62–Nrf2–antioxidant response element axis and eventually trigger cell death.

## Introduction

Nuclear factor erythroid 2-related factor 2 (Nrf2) is an oxidative response pathway protein, which can counterbalance intracellular reactive oxygen species (ROS) and protect cells from free radical stress-induced death [1]. Under normal conditions, the canonical Nrf2 pathway mediates the interaction of Nrf2 with Kelch-like ECH-associated protein 1 (Keap1), to the ubiquitination and constant proteasomal degradation of Nrf2. Under oxidative stress, the highly reactive thiol in Keap1 leads to conformational alterations and disassociation of Nrf2. Subsequently, Nrf2 translocates into the nucleus and binds to antioxidant response elements (AREs) by heterodimerization with small musculoaponeurotic fibrosarcoma (MAF) proteins (sMAFs) to activate the transcription of detoxifying and antioxidant genes. An important feature of the cytoprotective Keap1–Nrf2 system is that it is transient and inducible against intracellular redox disturbance resulting from environmental stimuli [2]. Nrf2 regulates various basal and inducible genes responsible for redox homeostasis, drug metabolism and excretion, iron metabolism, survival, proliferation, autophagy, proteasomal degradation, DNA repair, and mitochondrial physiology [3, 4]. Because of its protective role, the Nrf2 pathway generally prevents normal cells from chemical carcinogenesis via increases in the antioxidant and detoxification activities [5, 6]. Therefore, Nrf2 activation is essential for preventing cancer initiation and promoting anticancer activity [7]. However, cancer cells may undergo Nrf2-mediated aberrant metabolic adaptation to promote their survival.

Aberrant metabolic adaptation in cancer cells prevents the formation of a highly oxidized environment. In particular, upregulated expression of antioxidant scavenging molecules prevents ROS-induced death in cancer cells. This may limit the capacity of cancer cells to further adapt to additional ROS induction and lead to oxidative stress-dependent death, even with a slight ROS induction [8, 9]. Nrf2 plays a major role in the control of redox balance, which is crucial in cancer cells for various processes including tumor metabolism, aggressiveness, invasion, and metastasis. Persistent Nrf2 activation in cancer cells can lead to “Nrf2 addiction”, which is closely associated with drug resistance and malignancy [10, 11]. Accumulating evidence demonstrated that high levels of Nrf2 were commonly associated with poor prognosis in various cancer types, including glioma, non–small-cell lung cancer, lung adenocarcinoma, head and neck squamous cell carcinoma, breast cancer, hepatocellular carcinoma, pancreatic adenocarcinoma, gastric cancer, and colorectal cancer (CRC) [10]. The high Nrf2 signature was found to be associated with worsened disease-free and overall survival in 1360 patients with colorectal cancer from four independent datasets [12]. Nrf2 also drives metabolic reprogramming advantageous for cell proliferation in cooperation with other oncogenic pathways, such as those involving KRAS, BRAF, and MYC [13]. Therefore, the use of Nrf2-targeting cancer drugs may be a promising therapeutic approach for Nrf2-addicted colorectal cancer, where Nrf2 activation is aberrant.

Selenocompounds are prooxidant compounds with potential chemopreventive activities [14] against several major cancers, including prostate, lung, colon, and liver cancers [15–18]. The differences in the basal redox levels between normal and cancer cells suggest that prooxidant compound-induced upregulation of cellular ROS specifically targets cancer cells [19]. Selenocompounds have redox-active features, demonstrating stronger selectivity toward cancer cells than toward normal cells. Several mechanisms have been reported to underlie the anticancer effects of selenocompounds, such as oxidative stress responses, cell-cycle arrest, and program cell death pathway [20]. However, the mechanisms underlying the selective anticancer activity of selenocompounds, discriminating between transformed and normal cells, remain unclear. We hypothesized that selective inhibition of the Nrf2 pathway in malignant cancers is critical for the anticancer effects of selenocompounds. However, the mechanisms underlying the selective targeting of Nrf2 in cancer cells by selenocompounds warrant elucidation. In this study, we demonstrated that the selenocompound l-selenocystine (SeC) has selective cytotoxicity in Nrf2-addicted CRC cells, but not in non–Nrf2-addicted MSCs and normal human colon cells. Activating Nrf2 with siRNA targeting *Keap1* increased the sensitivity and cytotoxicity of the less Nrf2-addicted C_2_BBe_1_ cells to SeC. By contrast, the cytotoxicity to SeC was alleviated in Nrf2-addicted WiDr cells transfected with *Nrf2*-targeting siRNA. Therefore, SeC was noted to selectively attack constitutively activated Nrf2-addicted cancer cells through the P62–Nrf2–ARE axis, reducing Nrf2 and autophagy pathway expression, finally triggering cell death.

## Results

### Cytotoxicity and oxidative stress induced by SeC in Nrf2-addicted CRC cells and non–Nrf2-addicted cells

**Fig. 1:**
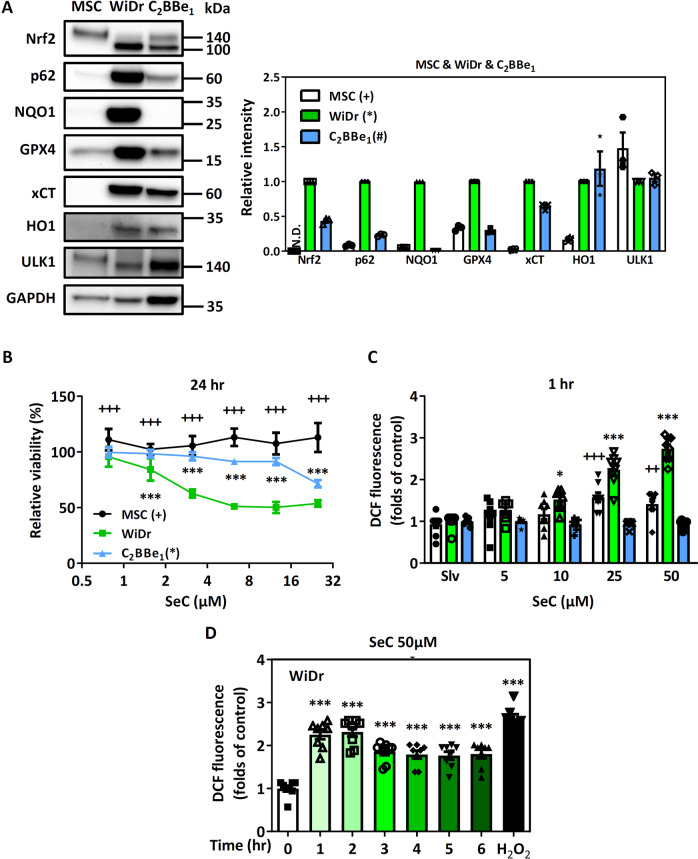
Cytotoxicity and oxidative stress induced by SeC in Nrf2-addicted and non–Nrf2-addicted cells. **A** Comparison of Nrf2-regulated protein levels among WiDr and C_2_BBe_1_ cells and MSCs. GAPDH was used as loading control (*n* = 3). The values were presented as average ± SD from three independent repeats. **B** Cytotoxicity of SeC in WiDr and C_2_BBe_1_ cells and MSCs after 24 h of treatment (*n* = 8). (* and ^+^*P* < 0.05, ** and ^++^*P* < 0.01^,^ *** and ^+++^*P* < 0.001, compared with same concentration of SeC in WiDr cells). **C** ROS levels after SeC treatment in WiDr and C_2_BBe_1_ cells and MSCs. **D** Time course of ROS production in WiDr cells after SeC treatment (*n* = 8). All quantified results of cytotoxicity and ROS were express as mean ± SD from eight independent repeats. (* and ^+^*P* < 0.05, ** and ^++^*P* < 0.01, *** and ^+++^*P* < 0.001, *n* = 8, compared with the solvent control group). Significance was determined by an unpaired *t* test.

In cancer cells, Nrf2 activation not only promotes progression and metastasis [21], but also induces chemotherapy and radiotherapy resistance [22]. The high Nrf2 signature is associated with worsened disease-free survival and overall survival in CRC patients. In the current study, we used two colorectal carcinoma cell lines, WiDr and C_2_BBe_1_ cells, as well as mesenchymal stem cells (MSCs) to elucidate Nrf2-target protein expression [23, 24]. WiDr cells appeared to have the highest level of Nrf2 (~105 kDa) and Nrf2-regulated proteins, such as p62 (SQSTM1), NQO1, GPX4, xCT (SLC7A11), HO1 and ULK1. The MSCs demonstrated the lowest levels of Nrf2 and Nrf2-regulated proteins. Mikac et al. detected two different molecular weight of Nrf2 (105 and 130 kDa) in two lung cancer cell lines by using western blotting analysis [25]. 105 kDa of Nrf2 is a stable form that may be associated with the constitutive activity of Nrf2 in lung cancer cells. These results indicated that the WiDr cells were Nrf2 addicted; while the MSCs acted similarly to the non–Nrf2-addicted normal cells. The C_2_BBe_1_ cells, expressed a lower level of Nrf2 and Nrf2-regulated protein than did the WiDr cells, noted as moderate Nrf2-addicted cells (Fig. 1A).

Since Nrf2 is a vital transcription factor regulating intracellular redox homeostasis, we analyzed whether SeC demonstrates toxicity in Nrf2-addicted WiDr cells. We found that SeC induced higher cytotoxicity in the WiDr cells than in the C_2_BBe_1_ cells and MSCs (Fig. 1B). In fact, the WiDr cells had the lowest IC_50_ for SeC; thus, they were the most sensitive to SeC (Supplementary Fig. 1A). SeC-induced apoptosis in the WiDr and C_2_BBe_1_ cells was determined using the Muse Annexin V & Death Cell Kit (Supplementary Fig. 1B). After 1 h of treatment, SeC increased intracellular ROS production in the WiDr cells, but not in the MSCs and C_2_BBe_1_ cells (Fig. 1C). Increasing ROS levels were detected over various time courses of SeC treatment in the WiDr cells (Fig. 1D). Human normal colon epithelial cell line CCD841 CoN to SeC after 24 h SeC treatment was also examined and displayed similar cellular viability and ROS levels to that in the MSCs (Supplementary Fig. 2). Taken together, these results demonstrated that SeC induces cell death and oxidative stress in Nrf2-addicted CRC cells.

### Inhibition of nuclear translocation of Nrf2 and downstream proteins by SeC in Nrf2-addicted cells

**Fig. 2:**
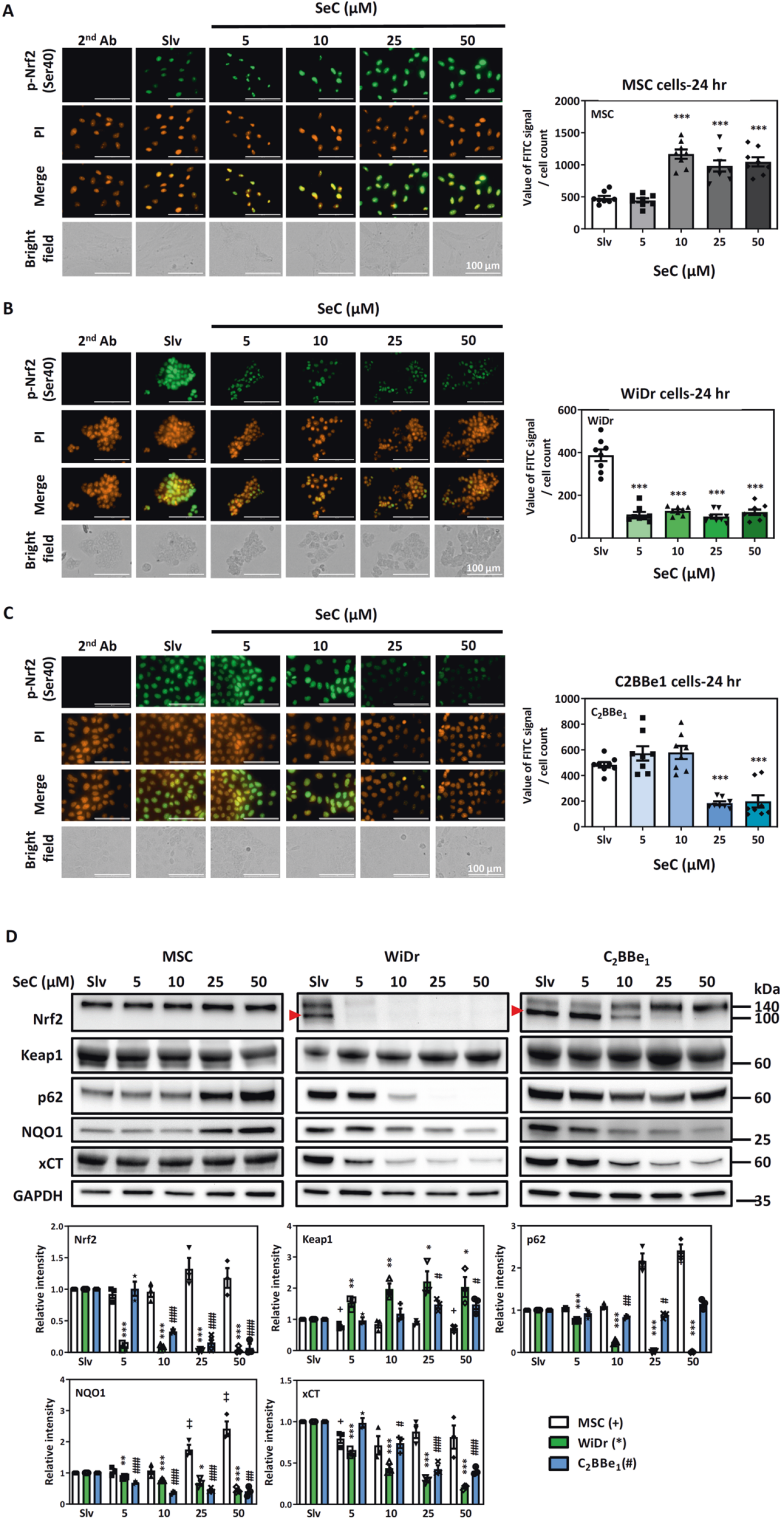
SeC inhibited nuclear translocation of Nrf2 and downstream proteins in Nrf2-addicted cells. **A**–**C** Translocation of phosphorylated Nrf2 into the nucleus in non–Nrf2-addicted MSCs (**A**) and WiDr (**B**) and C_2_BBe_1_ (**C**) cells. The cell count in field was determined using Image J. The fluorescence intensity was normalized with cell number from eight field. All quantified results are express as mean ± SD. (*P < 0.05, **P < 0.01, ***P < 0.001, compared with solvent control for each cell type). **D** Protein levels of NRF2, Keap1, p62, NQO1, and xCT in MSCs and WiDr and C_2_BBe_1_ cells after 24 h of SeC treatment. Relative levels of these target proteins were normalized to GAPDH (loading control. The values were presented as average ± SD from three independent repeats. (* and ^+^*P* < 0.05, ** and ^++^*P* < 0.01, ^*^** and ^+++^*P* < 0.001, *n* = 3, compared with solvent control for each cell type). Significance was determined by an unpaired *t* test.

Nrf2 translocates into the nucleus and then mediates various transcriptional responses of the cells to electrophile or ROS stimulation. We first determined whether SeC affects Nrf2 activation in the non-Nrf2-addicted MSCs and Nrf2-addicted cancer cells (Fig. 2). Our results demonstrated that SeC-induced Nrf2 activation and translocation in the non-Nrf2-addicted MSCs (Fig. 2A). However, SeC treatment inhibited Nrf2 activation in the Nrf2-addicted CRC cells (Fig. 2B, C).

Next, we investigated the Nrf2 pathway in the cells after 24 h of SeC treatment (Fig. 2D). SeC significantly inhibited the Nrf2 pathway, including Nrf2, p62, NQO1, and xCT, in the Nrf2-addicted WiDr cells. SeC decreased the lower molecular weight of Nrf2 (near 105 kDa) in Nrf2-addicted CRC cells. Higher Keap1 levels in the WiDr cells also indicated that Nrf2 is repressed after SeC treatment. As for C_2_BBe_1_ cells, which exhibited lower Nrf2 levels and less sensitivity to SeC, SeC also had an inhibitory effect on its Nrf2 pathway. On the contrary, SeC treatment increased p62 and NQO1 levels and reduced Keap1 levels in MSCs, and had no effects on the Nrf2 and xCT levels. Therefore, SeC does not inhibit the Nrf2 pathway in non-Nrf2-addicted MSCs.

We also examined the effects of SeC on the mRNA levels of *Nrf2 (NFE2L2)*, *KEAP1*, *NQO1*, *SQSTM1*, and *SLC7A11* in Nrf2-addicted CRC cells (Supplementary Fig. 3A, B) and non-Nrf2-addicted MSCs (Supplementary Fig. 3C) after 6, 12, or 24 h treatment. The mRNA levels of *NFE2L2*, *NQO1*, *SQSTM1*, and *SLC7A11* were not decreased, but rather increased after 6 h or 12 h of SeC treatment in WiDr and C_2_BBe_1_ cells. The increased mRNA levels of *NFE2L2* and *Nrf2*-target genes in SeC-treated MSCs were much higher than in Nrf2-addicted CRC cells (Supplementary Fig. 3). These results indicated that SeC may selectively inhibit Nrf2 pathway through post-transcriptional and/or post-translational mechanisms. Stability/turnover of mRNA is a vital mechanism for post-transcriptional control of gene expression [26]. We determined whether SeC treatment affects *Nrf2* mRNA stability by blocking de novo mRNA transcription using actinomycin D, a transcription inhibitor (Supplementary Fig. 4A). The comparison of the *Nrf2* mRNA levels in SeC-treated WiDr cells, exposure to actinomycin D for 0, 1, or 3 h, suggested that SeC decreased Nrf2 mRNA stability.

The stability of Nrf2 protein is also mediated through translation and proteasome. Keap1 tightly regulated the activity of Nrf2 by ubiquitination and proteasome-dependent degradation [27]. Keap1 may undergo auto-ubiquitination to sustain Nrf2, which in turn sufficiently activate downstream genes against oxidative stressed. We used immunoprecipitation assay to clarify the ubiquitination levels of Keap1 and Nrf2 after SeC treatment for 24 h (Supplementary Fig. 4B, C). The ubiquitination of Keap1 decreased in WiDr cells of 24 h SeC treatment. However, SeC treatment increased levels of Nrf2 ubiquitination at 9 h. Moreover, Nrf2 is also regulated by the GSK3β/β-TrCP phosphorylation-dependent ubiquitination system [28, 29]. SeC treatment did not induce GSK3β Tyr216 phosphorylation, which is associated with GSK3β activity (Supplementary Fig. 4D). This result indicated that SeC may inhibit Nrf2 pathway in Nrf2-addicted CRC cells through post-translational modification of Keap1 proteins.

### SeC-induced cellular and mitochondrial superoxide production in CRC cells

**Fig. 3:**
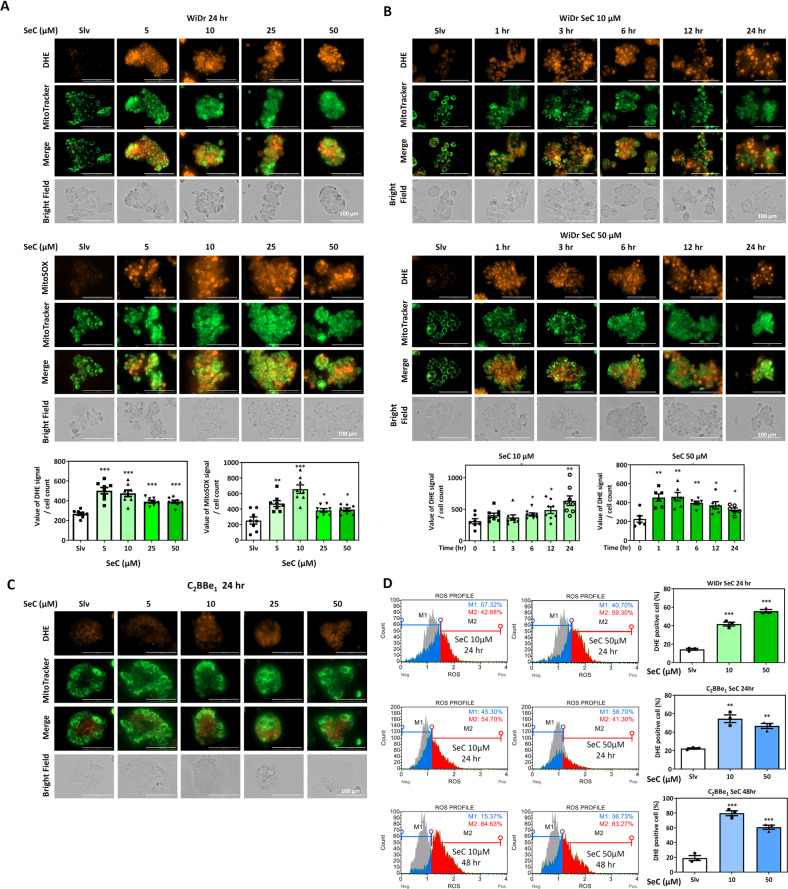
Superoxide production increased in Nrf2-addicted cells after SeC treatment. **A**, **B** Colocalization of DHE/MitoSOX and Mitotracker after exposure to various doses of SeC in Nrf2-addicted WiDr cells over different time courses. The cell count each in field was measured using Image J. The fluorescence intensity was normalized with cell number from eight fields. All quantified results are expressed as mean ± SD. (*P < 0.05, **P < 0.01, ***P < 0.001, compared with solvent control for each cell type). **C** DHE staining in C_2_BBe_1_ cells after 24 h of SeC treatment. **D** The percentage of DHE-positive cells determined through flow cytometry in SeC-treated WiDr and C_2_BBe_1_ cells (* and ^+^*P* < 0.05, ** and ^++^*P* < 0.01, ^*^** and ^+++^*P* < 0.001, *n* = 3, compared with solvent control for each cell type). Significa*n*ce was determined by an unpaired *t* test. The values were presented as average ± SD from three independent repeats.

The types of ROS include superoxide, hydrogen peroxide, and peroxynitrite. We used dihydroethidium (DHE) and its mitochondrion-targeted form MitoSOX for cellular and mitochondrial superoxide detection, respectively [30, 31]. Our results demonstrated that SeC induced considerable superoxide production in the Nrf2-addicted WiDr cells after 24 h of treatment (Fig. 3A). The colocalization of DHE red fluorescence and Mitotracker green fluorescence indicated that 24 h of SeC treatment induced cellular and mitochondrial superoxide.

We next determined superoxide production over various time courses in the SeC-treated WiDr cells (Fig. 3B) and found that after 1 h of treatment, the superoxide levels increased faster and to higher levels in the 50 μM SeC-treated Nrf2-addicted cells than in cells treated with 10 μM SeC. Therefore, SeC induced superoxide production in a dose-dependent manner. We also noted superoxide production in the C_2_BBe_1_ cells after SeC treatment for 24 h (Fig. 3C). DHE red fluorescent signals were slightly higher in the SeC treatment group than in the solvent control group. We investigated the superoxide production after SeC treatment in the WiDr and C_2_BBe_1_ cells through flow cytometry (Fig. 3D). The percentages of DHE-positive C_2_BBe_1_ cells revealed increased superoxide levels after 24 and 48 h of SeC treatment.

### Reduction in cytotoxicity to SeC by antioxidants in Nrf2-addicted WiDr cells

**Fig. 4:**
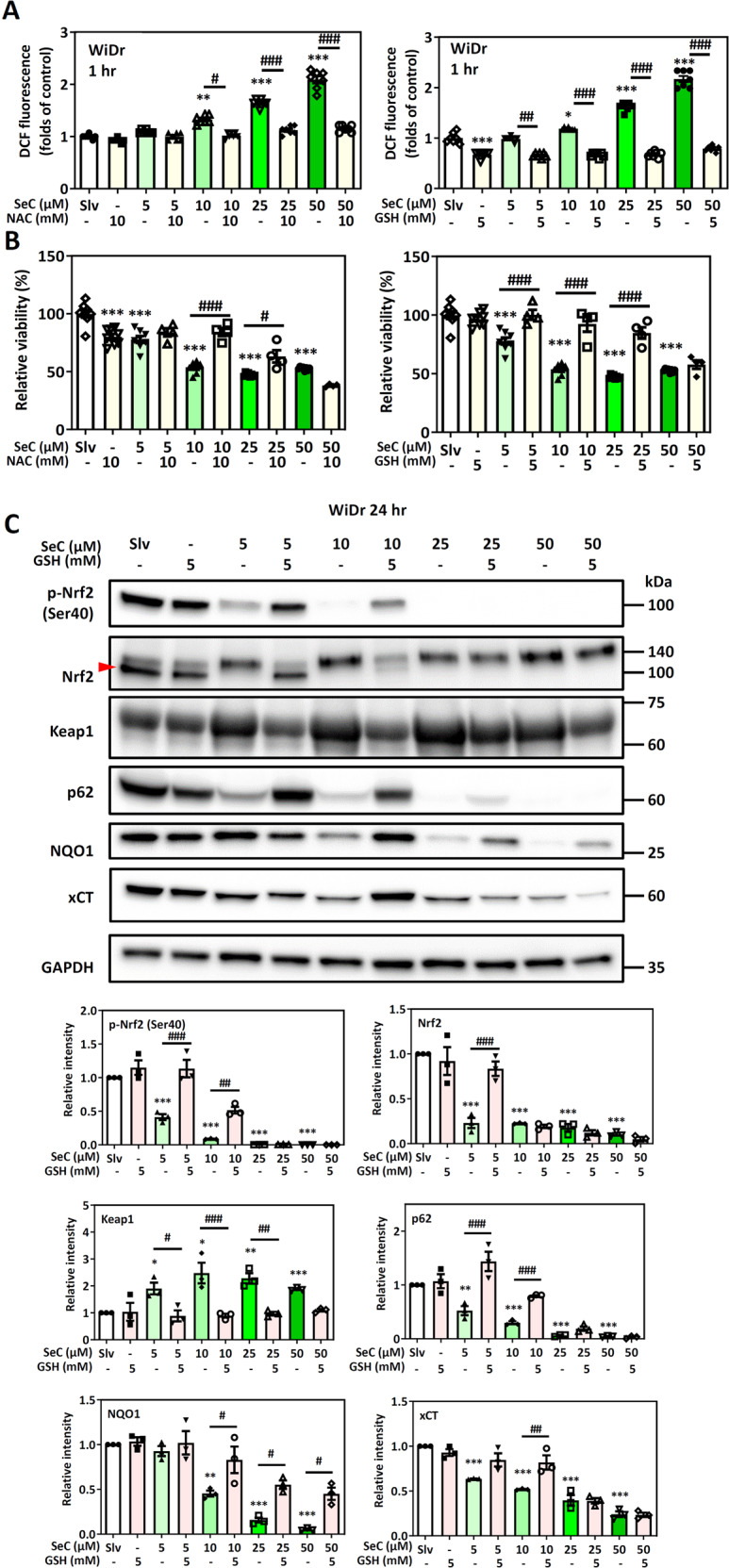
Effects of antioxidants on SeC-induced cell death and the Nrf2 pathway. **A** Intracellular ROS levels (*n* = 7) were measures by fluorescent reader after SeC treatment with or without antioxidants (NAC and GSH). **B** cell viability (*n* = 8) of WiDr cells exposed to SeC with or without antioxidants (NAC and GSH) pretreatment. The values were presented as average ± SD from seven or eirht independent repeats. **C** Levels of phosphorylated Nrf2 and Nrf2 regulated proteins in WiDr cells after SeC treatment and GSH. GAPDH was used as loading control (*n* = 3) (*P < 0.05, **P < 0.01, ***P < 0.001, compared with solvent control, ^##^*P* < 0.01, ^###^*P* < 0.001, compared with SeC-treated cells without antioxidant pretreatment, significance was determined by one-way ANOVA). The values were presented as average ± SD from three independent repeats.

Because SeC could increase ROS levels, we used the antioxidants N-acetylcysteine, (NAC) and glutathione (GSH) to clarify the role of oxidative stress in SeC-induced cell death. Antioxidant pretreatment reduced the levels of ROS in the WiDr cells after SeC treatment for 1 h (Fig. 4A). Cytotoxicity of the Nrf2-addicted WiDr cells to SeC was alleviated by antioxidant pretreatment. However, in the WiDr cells, the GSH could not rescue cell viability after 50 μM SeC treatment (Fig. 4B).

Next, we evaluated the effects of the antioxidants on the Nrf2 signal pathway in SeC-treated cells (Fig. 4C). Our results demonstrated that after 24 h of treatment, GSH partially reduced the inhibition effect of SeC on the Nrf2 pathways, including increased levels of Nrf2 phosphorylated at Ser40, p62, NQO1, and xCT and decreased levels of Keap1. NAC pretreatment has a similar effect on the SeC-treated WiDr cells (Supplementary Fig. 5). GSH could not inhibit cell death and rescue Nrf2 pathway inhibition after 50 μM SeC treatment. As aforementioned, Nrf2 levels were lower in the C_2_BBe_1_ cells than in the WiDr cells, demonstrating that SeC had lower cytotoxicity in the C_2_BBe_1_ cells. GSH had no significant effect on SeC-treated C_2_BBe_1_ cell viability after 24 h of treatment (Supplementary Fig. 6). This result indicated that in addition to ROS, SeC may induce death in Nrf2-addicted cancer cells through other metabolic pathways regulated by Nrf2.

### Dependence of Nrf2-addicted CRC cell survival during SeC treatment on autophagy

**Fig. 5:**
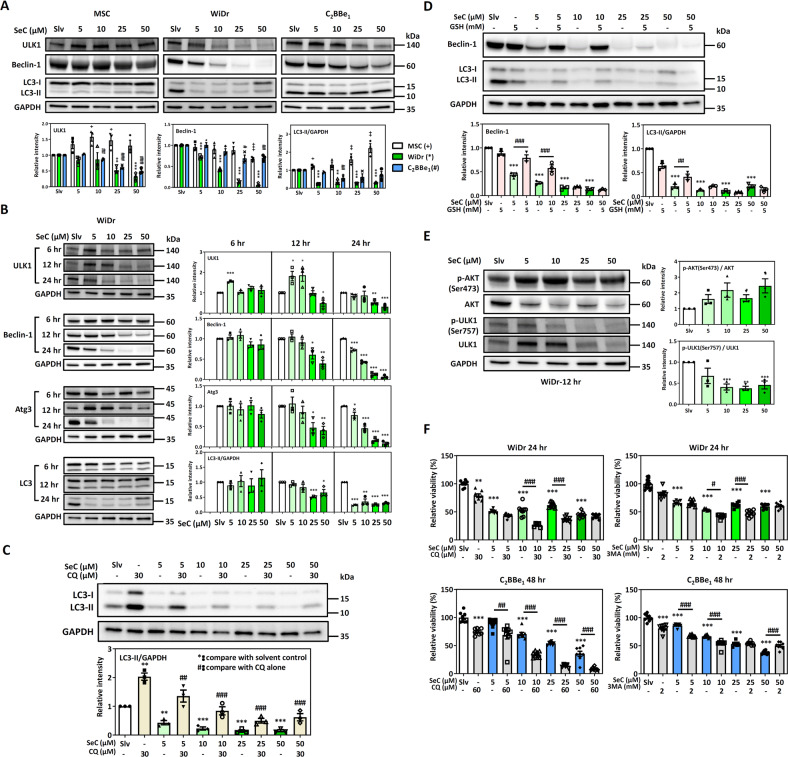
SeC inhibited autophagy in Nrf2-addicted CRC cells. **A** Protein levels of ULK1, Beclin-1 and LC3-II levels in MSCs, WiDr cells, and C_2_BBe_1_ cells after SeC treatment for 24 h. The values were presented as average ± SD from three independent repeats. (^+^, *, ^#^*P* < 0.05, ^++^, **, ^##^*P* < 0.01, ^+++^, ***, ^###^*P* < 0.001, compared with solvent control for each cell type, Significance was determined by an unpaired *t* test). **B** Autophagy proteins in SeC-treated WiDr cells after 6, 12, and 24 h The values were presented as average ± SD from three independent repeats. (*P < 0.05, **P < 0.01, ***P < 0.001, compared with solvent control, Significance was determined by an unpaired *t* test.). **C** LC3II were measured in the presence of CQ to determine autophagy flux in SeC-treated WiDr cells after 24 h. The values were presented as average ± SD from three independent repeats. (**P < 0.01, ***P < 0.001, compared with solvent control; ^##^*P* < 0.01, ^###^*P* < 0.001, compared with CQ alone). **D** Effects of GSH on the autophagy pathway in WiDr cells after SeC treatment for 24 h. Protein levels of Beclin-1 and LC3II were measured by immunoblotting. The values were presented as average ± SD from three independent repeats. (***P < 0.001, compared with solvent control; ^##^*P* < 0.01, ^###^*P* < 0.001, compared with SeC treatment without GSH pretreatment, significance was determined by one-way ANOVA). **E** AKT and ULK1 phosphorylation in SeC-treated WiDr cells after 12 h. GAPDH was used as loading control (*n* = 3). The values were presented as average ± SD from three independent repeats. **F** Cell viability of WiDr cells and C_2_BBe_1_ cells after SeC treatment with or without CQ or 3-MA pretreatment (*n* = 8). The values were presented as average ± SD from eight independent repeats. (**P < 0.01, ***P < 0.001, compared with solvent control; ^##^*P* < 0.01, ^###^*P* < 0.001, compared with SeC treatment without CQ pretreatment, significance was determined by one-way ANOVA).

Different stages of tumor progression, such as tumor initiation and expansion, invasion, metastasis, and resistance to therapy, involve autophagy [32]. Moreover, autophagy protein p62 sequester Keap1 leads to Nrf2 activation. Here, we determined the role of autophagy in Nrf2-addicted CRC cell death induced by SeC. Compared with non-Nrf2-addicted MSCs, the WiDr and C_2_BBe_1_ cells demonstrated decreased expression of autophagy pathway proteins after 24 h of SeC treatment. ULK1, Beclin-1, and LC3II levels demonstrated the most significant decrease in the SeC-treated WiDr cells. By contrast, ULK1 and LC3II increased in the SeC-treated MSCs. These results suggested that autophagy plays a protective role in cell survival after SeC treatment. Because autophagic flux is a dynamic, multiple-degradative process, we determined autophagy pathway protein expression at various time courses of SeC treatment in the Nrf2-addicted WiDr cells. Although ULK1 levels increased at 6 and 12 h after low-dose SeC treatment, the levels of Beclin-1, Atg3, and LC3 decreased after 12 h of SeC treatment (Fig. 5B). We then used chloroquine (CQ) to validate whether autophagic flux was inhibited by SeC treatment. CQ alone- group, used as the positive control, demonstrated autophagic flux blockage [33]. Compared with CQ alone, SeC did not increase LC3II in the presence of CQ pretreatment (Fig. 5C).

We also used GSH to determine the role of ROS in the autophagy pathway of the SeC-treated WiDr cells. ROS increase cytoprotective autophagy in cancer cells, leading to drug resistance and survival [34]. Our results demonstrated that Beclin-1 and LC3II levels increased in the presence of GSH and low-dose (5 and 10 μM) SeC treatment for 24 h. GSH pretreatment did not enhance autophagy proteins at a high SeC dose. AKT suppressed autophagy through the activation of mTOR, inhibiting the autophagy-initiating ULK1 kinase complex [35]. We further determined whether AKT is involved in the upstream pathway of autophagy inhibition by SeC in the Nrf2-addicted CRC cells. Increased AKT phosphorylation at Ser473 and decreased ULK1 phosphorylation at Ser757 were occurred after 12 h of SeC treatment (Fig. 5E). Furthermore, we used the autophagy inhibitors CQ and 3-methyladenine (3-MA) to investigate whether autophagy has a protective role in Nrf2-addicted CRC cells. We observed that the blockage of autophagy increased SeC cytotoxicity in the WiDr and C_2_BBe_1_ cells (Fig. 5F). Taken together, these results indicated that SeC inhibits autophagy activation resulting in Nrf2-addicted CRC cell death through the ROS-dependent and AKT/mTOR-ULK1 pathways.

### Increased SeC sensitivity in Nrf2-addicted CRC cells

**Fig. 6:**
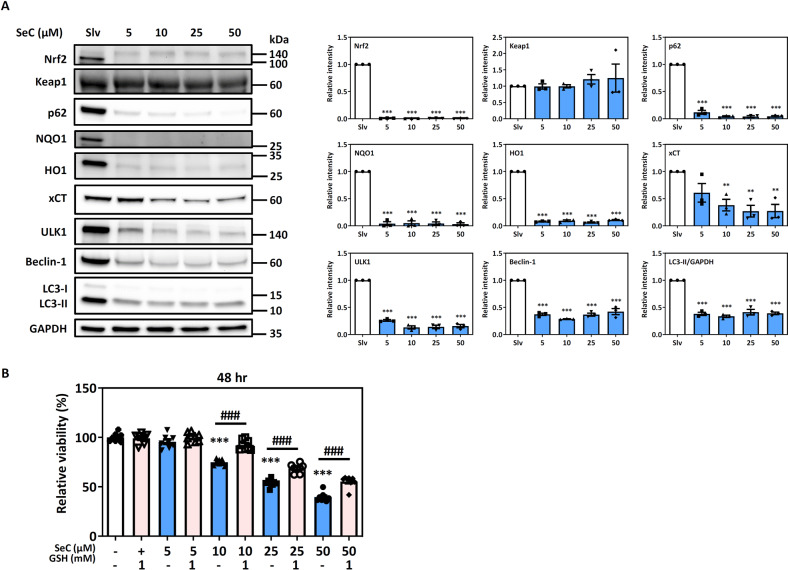
SeC inhibits Nrf2 and autophagy pathway proteins in C_2_BBe_1_ cells after 48 h of treatment. **A** Nrf2, Keap1, p62, NQO1, HO1, xCT, ULK1, Beclin-1, and LC3II levels in C_2_BBe_1_ cells after SeC treatment for 48 h (*p < 0.05, *p < 0.01, ***p < 0.001, compared with solvent control, significance was determined by an unpaired *t* test). The values were presented as average ± SD from three independent repeats. **B** Relative viability of SeC-treated C_2_BBe_1_ cells with or without GSH pretreatment for 48 h. The values were presented as average ± SD from eight independent repeats. (*p < 0.05, **p < 0.01, ***p < 0.001, compared with solvent control; ^##^*p* < 0.01, ^###^*p* < 0.001, compared with SeC alone, significance was determined by one-way ANOVA).

Compared with the Nrf2-addicted WiDr cells, the C_2_BBe_1_ cells expressed lower Nrf2-regulated protein levels and higher IC_50_ of SeC. However, 30% of the apoptosis occurred in the C_2_BBe_1_ cells after SeC treatment for 24 h. We further determined whether Nrf2 and autophagy pathway proteins are affected by 48 h of SeC treatment in the C_2_BBe_1_ cells (Fig. 6). We noted that SeC inhibited Nrf2 and autophagy pathway proteins, including Nrf2, p62, NQO1, HO1, xCT, ULK1, Beclin-1, and LC3II, and that superoxide accumulation occurred in the SeC-treated C_2_BBe_1_ cells after 24 and 48 h (Fig. 3D). Moreover, GSH could partially enhance cell viability in the C_2_BBe_1_ cells after SeC treatment for 48 h (Fig. 6B). These results indicated that the sensitivity of CRC cells to SeC may be associated with their Nrf2 addiction status.

**Fig. 7:**
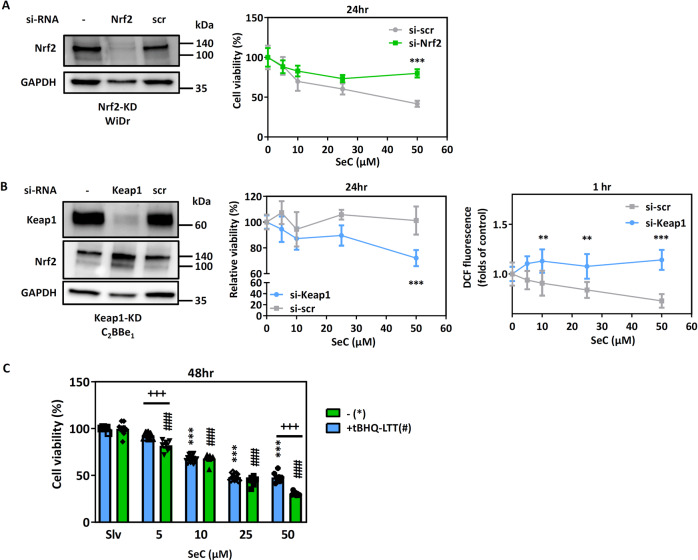
Effects of basal Nrf2 status on SeC cytotoxicity in CRC cells. **A** siRNA targeting scramble control (scr) and *Nrf2* transfected into WiDr cells to alleviate their Nrf2 addiction status. The relative viability determined using the MTT assay (*n* = 5). The values were presented as average ± SD from five independent repeats. **B** siRNA targeting scr and *Keap1* transfected into C_2_BBe_1_ cells to increase Nrf2 addiction status. The relative viability was determined using the MTT assay (*n* = 4). The ROS levels was measured after 1 h of SeC treatment (*n* = 5). The values were presented as average ± SD from four or five independent repeats. (*p < 0.05, *p < 0.01, ***p < 0.001, compared with scr). **C** Persist treatment with 50 μM tBHQ for 2 weeks to mimic Nrf2 overactivation in C_2_BBe_1_ cells. The relative viability was determined after SeC treatment for 48 h using the MTT assay (*n* = 8). The values were presented as average ± SD from eight independent repeats. (*p < 0.05, **p < 0.01, ***p < 0.001, compared with solvent control; ^##^*p* < 0.01, ^###^*p* < 0.001, compared with SeC alone). Significance was determined by two-way ANOVA.

To validate this hypothesis, we employed *Nrf2*-targeting siRNA transfection to reduce persistent Nrf2 activation in the WiDr cells (Fig. 7A). *Nrf2*-targeting siRNA transfection led to the alleviation of SeC cytotoxicity in the WiDr cells. In addition, Nrf2 pathway activation in the *KEAP1*-knockdown C_2_BBe_1_ cells increased their SeC sensitivity and cytotoxicity (Fig. 7B). We also used tert-butylhydroquinone (tBHQ), a Nrf2 activator, to persistently activate Nrf2 in the C_2_BBe_1_ cells for 2 weeks. The cytotoxicity of SeC in the C_2_BBe_1_ cells partially increased after tBHQ pretreatment for 2 weeks (Fig. 7C).

## Discussion

Constitutive activation of Nrf2 is strongly associated with cell survival in various cancers, and metabolic reprogramming is beneficial for cell proliferation [10]. CRC is one of the most common cancers affected by the interplay of environmental and genetic factors. The appropriate basal function of Nrf2 and Keap1 is essential for oncogenic process prevention in the colon; however, Nrf2 overactivation, resulting from Nrf2-induced inflammation and resistance to chemotherapy, increases colon cancer risk [36]. Moreover, the high Nrf2 signature has been noted to worsen disease-free and overall survival in patients with CRC [12]. In CRC, Nrf2 overactivation leads to Nrf2 addiction, demonstrated by malignant CRC phenotypes, which have poor prognosis. Therefore, the use of Nrf2-targeting drugs may be a promising therapeutic approach for treating Nrf2-addicted CRC.

Various selenocompounds, including inorganic and organic molecules with selenoamino acid and sodium selenite, have been used in cancer chemotherapy. Accumulating evidence suggests that Se-containing amino acids (e.g., SeC, selenomethionine, and methylselenocysteine) typically function as prooxidant agents with anticancer activity. Selenomethionine and methylselenocysteine have been reported to exhibit antiproliferative activity in human breast cancer cells [37, 38]. SeC is the most stable dimeric selenocompound, formed through diselenide oxidation of selenocysteine (SeCys) [39]. SeCys can be found in many selenoproteins, which have a homeostatic effect on cell physiological responses [39]. SeC has been reported to have a selective antiproliferative activity in melanoma and cervical cancer cells [17, 39, 40]. We previously reported that SeC selectively induces oxidative-mediated DNA damage by impairing homologous recombination repair of DNA double-strand breaks in hepatoma cells [41]. However, the mechanism by which SeC discriminates between transformed and normal cells remains unclear; in particular, the mechanism underlying the selective targeting of Nrf2 by SeC in cancer cells warrants elucidation.

**Fig. 8:**
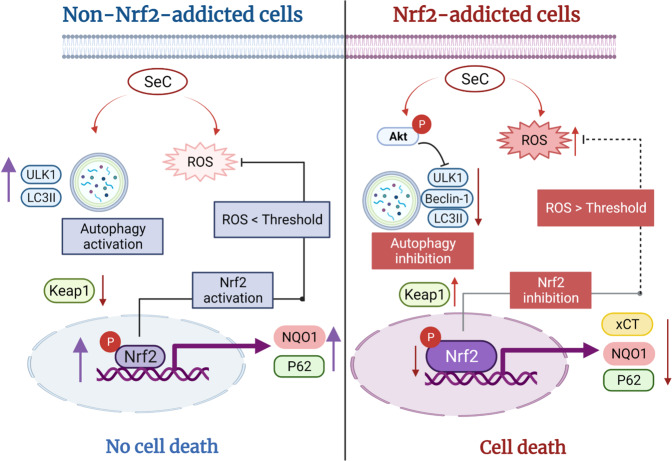
Selective inhibition of Nrf2 and autophagy due to SeC in Nrf2-addicted CRC cells. In non–Nrf2-addicted cells, SeC induced slightly low ROS levels and then effectively triggered Nrf2 and autophagy activations. The autophagic proteins, such as ULK1 and LC3II, increased in the SeC-treated non-Nrf2-addicted cells. SeC induced Nrf2 activation and translocation, resulting in NQO1 and HO-1 increasing in the non–Nrf2-addicted cells. Therefore, the ROS was below the threshold level and no cell death occurred. By contrast, SeC induced ROS prodiction and inhibited the Nrf2 and autophagy pathways in Nrf2-addicted cells. The antioxidative enzymes xCT and NQO1 were decreased by SeC. SeC inhibits Nrf2 activation, which may lead to anoverwhelming ROS accumulation, subsequently triggering cell death. P phosphorylation.

In this study, we observed that SeC exhibits selective cytotoxicity in the Nrf2-addicted CRC cell line WiDr by inhibiting the Nrf2 and autophagy pathways. First, we analyzed and compared the basal Nrf2 and Nrf2-regulated protein levels in Nrf2-addicted CRCs (WiDr and C_2_BBe_1_), with non Nrf2-addicted cells (MSCs and CCD841 CoN). Human MSCs and normal colon cells CCD841 CoN used as control cells. MSCs have been widely studied for clinical applications. Although human MSCs exhibit the multipotent ability to differentiate into various cell types under appropriate culture conditions, they rarely undergo spontaneous undergo malignant transformation [42]. Here, we found that MSCs and CCD841 CoN cells expressed basal (limited) Nrf2 levels in normal culture conditions. By contrast, the two CRC cell lines WiDr and C_2_BBe_1_ expressed higher Nrf2 and Nrf2-regulated protein levels than did the MSCs or CCD841 CoN cells. Of the two CRC cell lines, the WiDr cells demonstrated considerably persistent activation of Nrf2 characteristics in basal expression levels. Moreover, SeC was noted to have great potential to specifically target Nrf2-addicted CRC cells (Fig. 8). In non-Nrf2-addicted cells (e.g., MSCs and CCD841 CoN cells), SeC induced slightly low ROS levels and then effectively triggered Nrf2 and autophagy defense responses. Nrf2 activation in the normal cells prevented ROS-induced cell death. However, SeC selectively inhibited Nrf2 and autophagy pathway proteins in Nrf2-addicted CRC cells, leading to ROS-induced cell death.

Our results demonstrated that SeC cytotoxicity considerably differed between Nrf2-addicted and non–Nrf2-addicted cells. SeC did not cause considerable cytotoxicity in MSCs or CCD841 CoN cells. We hypothesized that the sensitivity to SeC in different cancer cells may be closely associated with the cellular status of Nrf2 addiction. WiDr cells express a sable, non-canonically regulated Nrf2 form. This shorter and stable 105 kDa Nrf2 form may closely associated with the constitutive activation of Nrf2 in tumor cells [25]. Previous RNA-seq data showed that six different *Nrf2* transcripts expressed in A549 cells. Shorter Nrf2 form may originate from an alternative transcription or translation which disturbs expression or lacks Keap1 binding motifs [25]. We compared Nrf2 proteins in *Nrf2* and *Keap1* siRNA cells by westering blotting. Both 105 and 140 kDa signals were increased in *Keap1*-knockdown C_2_BBe_1_ cells; however, both 105 and 140 kDa signals were decreased in *Nrf2*-knockdown WiDr cells. These results indicated that 105 and 140 kDa are Nrf2-specific.

The IC_50_ of SeC was 71 μM in WiDr cells and 167 μM in C_2_BBe_1_ cells after 24 h of treatment. The relative Nrf2 levels in C_2_BBe_1_ were 0.43-fold those in WiDr cells. This result indicated that C_2_BBe_1_ cells exhibited lower levels of Nrf2 addiction than the WiDr cells. The percentage of apoptosis was also higher in SeC-treated WiDr. To confirm this hypothesis, we used specific *Nrf2*-targeting siRNA transfection to decrease levels of Nrf2 activation. The cytotoxicity of SeC was alleviated after *Nrf2*-targeting siRNA transfection in WiDr cells, similar to the result in SeC-treated C_2_BBe_1_ cells. Conversely, activation of the Nrf2 pathway through Keap1 knockdown in C_2_BBe_1_ cells increased the sensitivity and cytotoxicity of C_2_BBe_1_ cells to SeC.

The mRNA levels of *NFE2L2*, *NQO1*, *SQSTM1*, and *SLC7A11* were not decreased, but rather increased after 6 h or 12 h of SeC treatment in WiDr and C_2_BBe_1_ cells. It is noted that the increased mRNA levels of *NFE2L2* and *NRF2*-target genes in SeC-treated MSCs were much higher than in Nrf2-addicted CRC cells. Meanwhile, SeC also decreased *NFE2L2* mRNA stability. We speculated that SeC may selectively inhibit Nrf2 pathway through post-transcriptional control or/and post-translational mechanisms. Oxidative stress has an impact on mRNA stability via the regulation of mRNA binding protein and miRNA machinery [43]. The detailed mechanism of SeC in decreasing mRNA stability requires further experiments. Nrf2 is negatively regulated by Keap1 for ubiquitination and proteasome-dependent degradation in normal homeostatic conditions. It is speculated that Keap1 auto-ubiquitination may be essential for Nrf2 accumulation to trigger antioxidant response. Upon oxidative stress response, electrophilic or ROS react with cysteine residues in Keap1 leading to the conformational changes of Keap1-E3 ligase complex. Overexpression of Keap1 suppresses the nuclear translocation and transcriptional activity of Nrf2. In addition to Keap1-E3 ligase, Nrf2 activation is sophisticatedly mediated by a variety of mechanisms, post-translational modification, and cross talk with other signaling pathways [27]. For example, GSK3β/β-TrCP phosphorylation-dependent ubiquitination pathway plays a supplementary pathway for Nrf2 regulation. We found that Keap1 protein levels increased after SeC treatment in WiDr cells. Thus, SeC not only triggered Nrf2 mRNA instability, but also decreased levels of auto-ubiquitination in Keap1 protein. However, the GSK3β may not involve in the effect of SeC in the selective inhibition of Nrf2. These effects induced by SeC may lead to the inhibition of Nrf2 in Nrf2-addicted CRC cells. The detailed molecular mechanism underlying the targeting of Nrf2 by SeC needs further investigation.

Selenocompound could be prooxidants which directly increase levels of ROS. Our result showed that SeC indeed increased higher ROS levels in WiDr cells than in MSC cells after 1 h treatment. Increased ROS normally activated Nrf2 pathway in non- Nrf2-addicted MSC cells. However, SeC constantly induced superoxide production and inhibited Nrf2 pathway after 24 h treatment in WiDr cells. Nrf2 pathway is important in upregulating antioxidant and cytoprotective genes against oxidative and electrophilic stress. Our results indicated that in Nrf2-addicted cancer cells, SeC inhibits Nrf2 activation, which may lead to an overwhelming ROS accumulation, subsequently triggering cell death. Antioxidants can reduce the cytotoxicity of SeC in Nrf2-addicted cells. In canonical Keap1-Nrf2 pathways, Keap1 interacts with Nrf2 and Nrf2 is constantly ubiquitinated and degraded by 26 S proteasome. Under stress conditions, Keap1 is modified by ROS and dissociated with Nrf2, leading to Nrf2 activation. In the noncanonical Keap1-Nrf2 pathway, phosphorylated p62 competitively binds to Keap1, resulting in the escape of Nrf2 from Keap1 interaction and then degradation of Keap1 together with autophagic cargo. However, p62 is also one of the targets of Nrf2, indicating that a positive feedback loop is present in the p62-Keap1-Nrf2 axis [44]. Our result revealed that SeC increased levels of Keap1 and decreased autophagy protein p62 in WiDr cells after 24 h of treatment. Therefore, SeC may inhibit the translocation of Nrf2 into the nucleus and interfere in the positive feedback loop p62-Keap1-Nrf2 axis of the noncanonical Keap1-Nrf2 pathway, resulting in the elevation of intracellular ROS above a critical threshold. In addition to ROS, SeC may also induce cell death of Nrf2-addicted cancer cells through other metabolic pathways regulated by Nrf2. Constitutive activation of Nrf2 in cancer cells induces prosurvival genes and promotes cellular proliferation through metabolic reprogramming and repression of cancer cell apoptosis [45]. Several metabolic reprogramming pathways are regulated by Nrf2, including NADPH generation, pentose phosphate pathway, fatty acid synthesis and oxidation, purine biosynthesis, and other transcription factors [45]. Whether SeC affects the metabolic pathway in Nrf2-addicted cancer cells requires further clarification.

The autophagy and Keap1-Nrf2 pathways constitute the major cellular defense systems against metabolic and oxidative stress. These two pathways can crosstalk through p62/SQSTM1. P62 mediates the autophagic degradation of polyubiquitinated substrates through interactions with LC3 on autophagosomes [46]. Towers et al. reported that the reduction of autophagy results in growth suppression in various cancers driven by *KRAS* or *BRAF* mutations, including liver, pancreatic, lung, breast, and colon cancers [47]. Our result also demonstrated that SeC reduced ULK1, Beclin-1, and LC3II expression in the WiDr cells. Moreover, autophagic flux was inhibited by SeC treatment. GSH pretreatment followed by 25 and 50 μM SeC treatment did not increase autophagy protein levels. GSH scavenges ROS directly or indirectly, and limited the oxidative signal in the cells [48]. Notably, GSH acts as a substrate for several antioxidant enzymes [49]. Direct inhibition of Nrf2 activation by SeC reduced the efficacy of GSH against SeC-induced ROS overproduction. Moreover, ROS may play a dual role in autophagy induction and inhibition. For instance, ROS may trigger transcription factor activation to induce autophagy-related gene expression. By contrast, ROS oxidize ATG proteins and PTEN to suppress autophagy [50]. In the current study, increased AKT phosphorylation at Ser473 and decreased ULK1 phosphorylation at Ser757 were noted after SeC treatment for 12 h. Our results also demonstrated that the blockage of autophagy increased SeC cytotoxicity in the WiDr and C_2_BBe_1_ cells. Moreover, SeC inhibited autophagy activation, resulting in the death of Nrf2-addicted CRC cells through the AKT/mTOR-ULK1 pathway.

In conclusion, SeC induces cytotoxicity selectively in Nrf2-addicted CRC cells by blocking Nrf2 and autophagy pathways. SeC may inhibit Nrf2 activation and then interfere with the noncanonical positive feedback loop of the p62-Keap1-Nrf2 axis, resulting in an elevation of the intracellular ROS levels above a critical threshold. Moreover, SeC reduces autophagy protein expression via the AKT/mTOR-ULK1 pathway. Considering its selective cytotoxicity in Nrf2-addicted cancer cells, SeC has great potential for application in precision medicine for cancer therapy.

## Materials and methods

### Materials

NAC, GSH, 2ʹ,7ʹ-dichlorodihydrofluorescein diacetate (DCFH-DA), 3-MA, CQ, and tBHQ were purchased from Sigma-Aldrich. SeC was obtained from ACROS Organics (Thermo Fisher Scientific). SeC stock solution was dissolved in 1 N sodium hydroxide (Sigma-Aldrich) and then titrated with 1 N HCl to adjust pH. Solvent control was prepared with the same proportions of NaOH and HCl. The cells were treated with 5, 10, 25, and 50 μM SeC with or without pretreatment with the various inhibitors.

### Cell line and cell culture

The colorectal adenocarcinoma cell lines WiDr and C_2_BBe_1_ were obtained from BCRC (Bioresource Collection and Research Center, Food Industry Research and Development Institute, Taiwan). STR profile performed from BCRC. The human normal colon epithelial cell line CCD841 CoN was obtained from American Type Culture Collection (ATCC). STR profile performed from ATCC The MSCs were obtained as a gift from Dr. Chao-Ling Yao (National Cheng Kung University).

The WiDr cells were cultured in a medium containing minimum essential medium (MEM)-EAGLE Earle’s Salts Base (Biological Industries), penicillin (Hyclone), sodium pyruvate (PanReac AppliChem), and 10% fetal bovine serum (FBS; PEAK SERUM). The C_2_BBe_1_ cells were cultured in high-glucose DMEM (Biological Industries) with penicillin, sodium pyruvate, 10% FBS, and 0.01 mg/mL human transferrin (Sigma-Aldrich). The CCD841 CoN cells were cultured in ATCC-formulated Eagle’s MEM with 10% FBS. All the aforementioned cells were incubated at 37 °C in a humidified atmosphere with 5% CO_2_.

### Cell viability assay

The cells were seeded in 96-well plates at appropriate cell numbers per well. After SeC treatment for 24 h, the culture media were replaced with a new medium containing 0.5 mg/mL 3-(4,5-dimethylthiazol-2-yl)-2,5-diphenyltetrazolium bromide (MTT; Invitrogen, Molecular Probe), followed by incubation at 37 °C for 1 h. Next, the medium was removed from the wells, followed by the addition of dimethyl sulfoxide (DMSO) to dissolve crystalline MTT. Cell viability was then assessed at 535 nm on an ELISA reader (SpectraMax ABS; Molecular Devices).

### Intracellular ROS determination

The cells were added to 96-well black plates. After the indicated treatment, 25 μM H2DCF-DA (Sigma-Aldrich) was added to the wells. After 45 min, fluorescence was detected at Ex 485 nm/Em 525 nm on a fluorescent reader from BioTeK. The mitochondrial and cellular superoxide levels were measured using MitoSOX Red (Invitrogen, Molecular Probe, OR, USA) and DHE (Invitrogen).

The cells were cultured under a sterile coverslip until 60–70% confluency in a six-well culture plate. After SeC treatment, the cells were stained with 2 μM MitoSOX or DHE at 37 °C for 30 min. The cells were then washed three times with phosphate-buffered saline (PBS) to remove excess fluorescent dye; the fluorescence intensity was then determined using a fluorescence scanner (Lionheart; BioTeK) or Guava Muse Cell Analyzer at 580 nm for MitoSOX Red and 600 nm for DHE. The average fluorescent intensity was calculated as MitoSOX Red or DHE intensity divided by cell count in the field. The cell count in each field was determined using Image J.

### Apoptosis assay

The cells were cultured in 6-well plates and then treated with 5, 10, 25, and 50 μM SeC for 24 h. The percentage of apoptosis induced by SeC was determined using the Muse Annexin V & Death Cell Kit (Luminex), according to the manufacturer’s instructions. Apoptosis-positive cells were detected using a Guava Muse Cell Analyzer.

### Immunofluorescence staining

The cells were cultured under a sterile coverslip until 60–70% confluency in 6-well culture plates and then treated with SeC for 24 h. They were fixed and permeabilized using methanol. Anti-Nrf2 (phospho S40, ARG40667, 225×) antibody was obtained from Arigo Biolaboratories. Anti-rabbit IgG conjugated with Alexa Fluro 488 (Invitrogen, Molecular Probe) was used as the secondary antibody, and propidium iodide (PI) was used as the nuclear counterstain. Nrf2 phosphorylation at Ser40 was determined using a fluorescence scanner (Lionheart; BioTeK). The cell count in each field was determined using Image J.

### Western blot analysis

The cells were harvested using the M-PER lysis buffer (Thermo Fisher Scientific, Cat.78501, Grand Island, NY, USA) along with the phosphatase and protease inhibitor cocktail (Thermo Fisher Scientific, Cat.78442). After centrifugation at 14,000 rpm and 4 °C for 10 min, the supernatants were collected. The protein concentration in this mixture was determined using the BCA assay (Pierce Biotechnology, Cat.23225). Next, the supernatant was mixed with 4× sample buffer (Bio-Rad, #1610747). Equal amounts of protein were resolved using the TGX Stain-Free FastCast Acrylamine Kit (Bio-Rad, 7.5% Cat. #1610181, 10% Cat. #1610183, 12.5% Cat. #1610185) and transferred onto an Immuno-Blot PVDF Membrane (Bio-Rad, #1620177). After the membrane was blocked with 5% casein in PBS, it was probed with the designated first antibodies against AKT (Cell Signaling, #4685, 1000× dilution), phospho-AKT Ser473 (Cell Signaling, #4060, 1000× dilution), Beclin-1 (Cell Signaling, #3495, 1000× dilution), LC3 I/II (Cell Signaling, #12741, 1000x dilution), Atg3 (Cell Signaling, #3415, 100X), NRF2 (Arigo, ARG53382, 1000x dilution), phospho-NRF2 Ser40 (Arigo, ARG40667, 2000× dilution), SQSTM1/p62 (ABclonal, A7758, 2000× dilution), NQO1 (Cell signaling, #62262, 2500× dilution), GPX4 (Arigo, ARG41400, 1000× dilution), xCT (Arigo, ARG57998, 1000× dilution), HO1 (Cell Signaling, #43966, 1000× dilution), ULK1 (Cell Signaling, #8054, 1000x dilution), phospho-ULK1 Ser757 (Cell Signaling, #6888, 1000× dilution), Keap1 (Cell Signaling, #8047, 1000× dilution), GSK3β (ABclonal, A11731, 2000X), phospho-GSK3β-Y216 + GSK3α-Y279 (ABclonal, AP0261, 1000× dilution) and GAPDH (Merck, MAB374, 100,000× dilution), followed by incubation with secondary antibodies (horseradish peroxidase-conjugated anti-mouse or anti-rabbit antibodies). Anti-mouse and anti-rabbit antibodies were obtained from Thermo Fisher Scientific (anti-mouse Cat.31430, anti-rabbit Cat.31460, 100,000× dilution). Chemiluminescent signals were detected using Cytiva and TOPBIO MultiGel-21. The protein band intensity detected using QLIQS was used to measure protein expression.

### Quantitative real-time reverse transcription polymerase chain reaction assays

Cells were treated with SeC for 6, 12, 24 h, and RNA was then extracted using an RNAzol RT kit (Life Technologies, Rockville, MD, USA). The cDNA was synthesized using the total RNA (2 μg) by iScript^TM^ cDNA Synthesis kit (Bio-Rad). Real-time quantitative PCR was performed using QuantiNova™ SYBR® Green PCR (Qiagen, Valencia, CA) on CFX96 PCR instrument (Bio‑Rad Laboratories, Inc., Hercules, CA, USA). The thermocycling conditions were as follows: preamplification 2 min at 95 °C, PCRs were followed by 40 cycles of 5 s at 95 °C and 10 s at 60 °C. The relative expression of each gene was calculated by the relative fold change between treated and control groups using the 2^−ΔΔCq^ method. The PCR primer sequences used in this study were as follows. Human *GAPDH* forwards, 5′-TGCACCACCAACTGCTTAGC-3′, and reverse, 5′-GGCATGGACTGTGGTCATGA-3′; human *NRF2 (NFE2L2)* forwards, 5′-TCTGACTCCGGCATTTCACT-3′, and reverse, 5′-GGCACTGTCTAGCTCTTCCA-3′; human *KEAP1* forwards, 5′ CATCCACCCTAAGGTCATGGA-3′, and reverse, 5′-GACAGGTTGAAGAACTCCTCC-3′; human *p62 (SQSTM1)* forwards, 5′-TCCTGCAGACCAAGAACTATGACATCG-3′, and reverse, 5′- TCTACGCAAGCTTAACACAACTATGAGACA-3′; human *NQO1* forwards, 5′-CGCAGACCTTGTGATATTCCAG-3′, and reverse, 5′-CGTTTCTTCCATCCTTCCAGG-3′; human *xCT (SLC7A11)* forwards, 5′-CCCAGATATGCATCGTCCTT-3′, and reverse, 5′-GCAACCATGAAGAGGCATGT-3′.

### mRNA stability analysis

10 μg/mL of actinomycin D was used to determine mRNA stability by interfering transcription. WiDr cells were pretreated with SeC for 3 h, then treated with actinomycin D for 0, 1, or 3 h respectively followed by the RNA isolation. *Nrf2* mRNA relative levels were determined by normalized to *GAPDH* using the ΔCt method. The relative amount of *Nrf2* mRNA without actinomycin D treatment was set to 100% [51].

### Immunoprecipitation assay

The WiDr cells were treated with 50 μM SeC for 9 h or 24 h. The cell lysates were prepared in M-PER lysis buffer along with the phosphatase and protease inhibitor cocktail, and then centrifuged at 14,000 rpm for 15 min. Equal amounts of cell lysate were incubated with anti-Nrf2 (GeneTex, GTX103322, 1.3 μg) or anti-Keap1 antibody (ABclonal, A17061, 1 μg) at 4 °C for overnight. The immune complexes were precipitated with protein A-Sepharose beads at 4 °C for 4 h. The beads were washed with 0.2% Tween 20 in PBS and eluted in 150 mM glycine-HCl (pH2.6). The eluted proteins were neutralized by adding 1.7 M NaOH, and then boiled in 4× sample buffer. Proteins were separated by TGX Stain-Free FastCast Acrylamine Kit and immunoblotted with anti-ubiquitin antibody (Cell signaling, #58395, 1000× dilution).

### siRNA transfection

The WiDr and C_2_BBe_1_ cells were transfected with siRNA using Lipofectamine RNAiMAX Reagent (Invitrogen). *NFE2L2* human siRNA oligo duplex and universal scrambled negative control duplex were obtained from OriGene. siGENOME human *KEAP1* siRNA-smart pool and nontarget siRNA pool were purchased from Dharmacon siGENOME.

The cells were transfected with siRNA against *NRF2* or *KEAP1* or universal scrambled negative control siRNA for 48 h, according to the manufacturer’s instructions. After incubation, the transfection medium was replaced with normal culture medium, and the cells were treated with SeC for 24 h. Finally, cell viability was measured using the MTT assay.

### Statistical analysis

Statistical analyses were performed using Prism (version 9.0; GraphPad Software, San Diego, CA, USA). The differences between the treatment groups, analyzed using an unpaired *t* test and ANOVA, were considered statistically significant when their *P* values were <0.05, <0.01, or <0.001.

## Supplementary information

Supplementary Figures

Supplementary material

Checklist

## Data Availability

All data that supported the findings of this study are available from the corresponding author.
