# Supplementary material for: Blockage of Nrf2 and autophagy by L-selenocystine induces selective death in Nrf2-addicted colorectal cancer cells through p62-Keap-1-Nrf2 axis
Source: Cell Death Dis. 2022 Dec 20;13(12):1060. doi: 10.1038/s41419-022-05512-2 (PMC9768144; doi:10.1038/s41419-022-05512-2)
Supplement: Supplementary file 1 — Supplementary Figures [file 41419_2022_5512_MOESM1_ESM.docx]

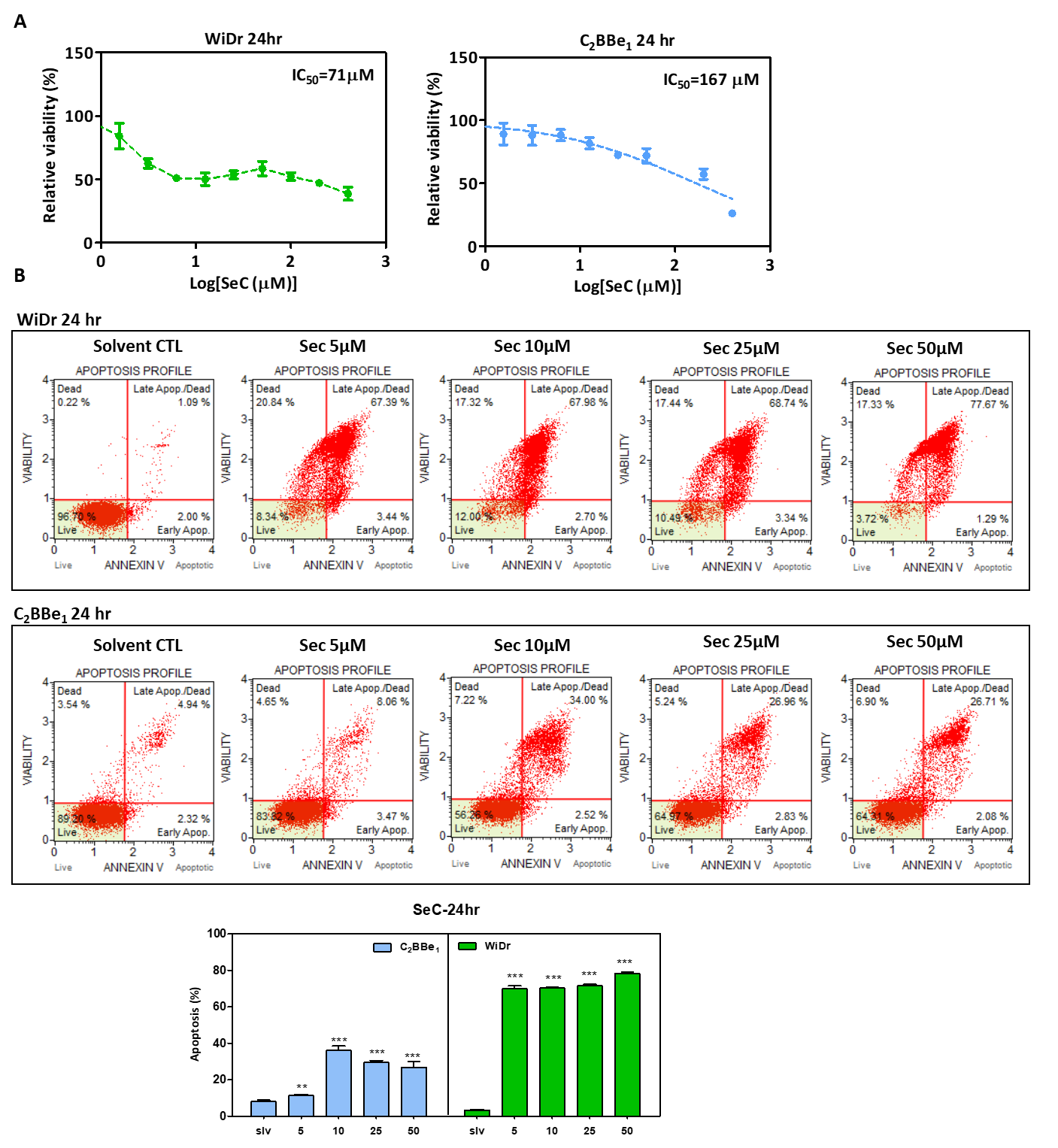


**Supplementary Fig. 1 IC_50_ and apoptosis in WiDr and C_2_BBe_1_ cells after SeC treatment. A** Cytotoxicity to SeC in WiDr and C2BBe1 cells after 24 h of treatment determined using the MTT assay. The nonlinear regression analysis of IC_50_ performed for log(inhibitor) vs. normalized response-variable slope in GraphPad Prism. **B** SeC-induced apoptosis in WiDr and C_2_BBe_1_ cells determined using the Muse Annexin V & Death Cell Kit. The values were presented as average ± SD from three independent repeats.


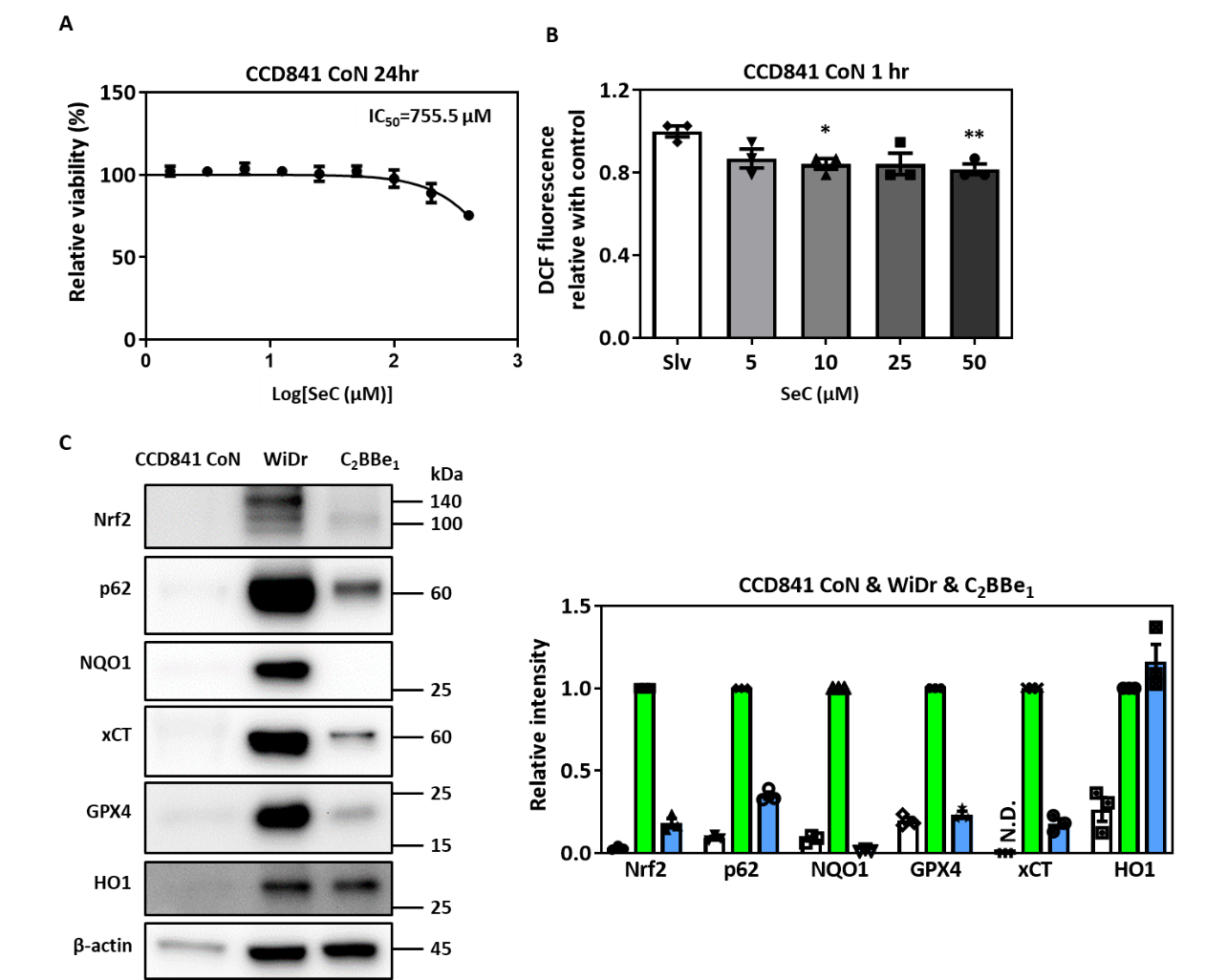


**Supplementary Fig. 2 Cytotoxicity to and oxidative stress induced by SeC in the human normal colon epithelial cell line CCD841 CoN. A** Cytotoxicity to SeC in CCD841 CoN cells after 24 h of treatment, determined using the MTT assay. The values were presented as average ± SD from eight independent repeats. **B** ROS levels after SeC treatment in CCD841 CoN cells after 1 h of treatment. **C** Comparison of NRF2-regulated protein levels among WiDr and C_2_BBe_1_ cells and CCD841 CoN. GAPDH was used as loading control. The values were presented as average ± SD from three independent repeats.


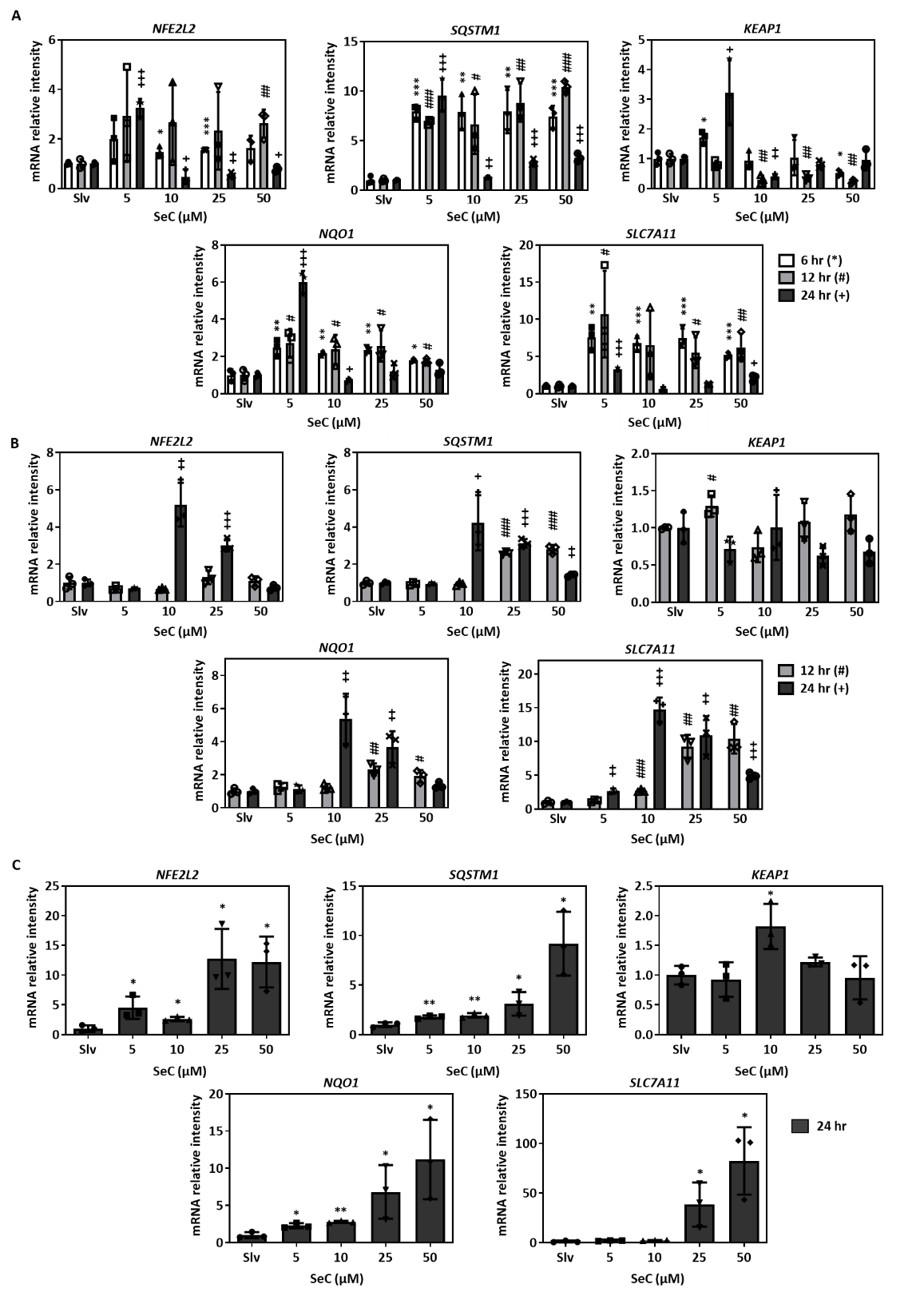


**Supplementary Fig. 3 Effects of SeC on *NRF2* and *NRF2*-target mRNA levels in CRC cells and MSCs after 6, 12 or 24 h treatment.** Quantification of the *Nrf2 (NFE2L2), p62 (SQSTM1), keap1, NQO1 and xCT (SLC7A11)* mRNA levels in **A** WiDr, **B** C_2_BBe_1_ cells and **C** MSCs cells after SeC treatment at 6, 12, or 24 h. *Nrf2* and *Nrf2* target mRNA levels were normalized to GAPDH mRNA levels. The values were presented as average ± SD from three independent repeats.


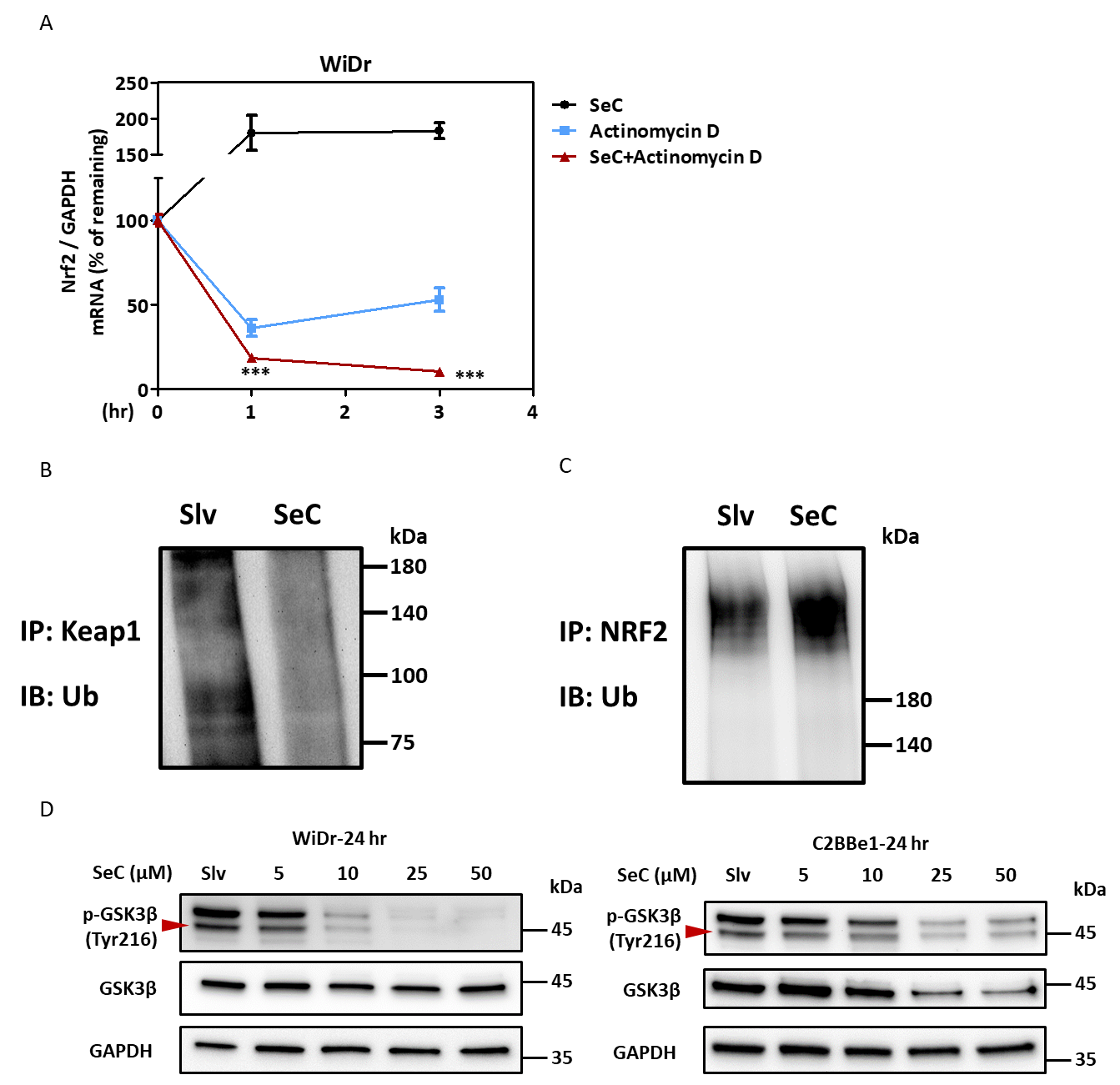


**Supplementary Fig. 4 Effect of SeC in *Nrf2* mRNA stability, ubiquitination of Keap1 and NRF2, and GSK3β phosphorylation in WiDr cell. A.** *Nrf2* mRNA stability in SeC-treated cells. WiDr cells were pretreated with 50 μM of SeC for 3 h, and then actinomycin D (10 μg/mL) for 0, 1, or 3 h and collected for RNA isolation. The experiment was performed with three repetitive treatments. *Nrf2* mRNA relative levels were determined by normalized to GAPDH using the ΔCt method. The relative amount of *Nrf2* mRNA without actinomycin D treatment was set to 100%. *** *P < 0.001*, compared with SeC treatment group. **B** Ubiquitination of Keap1 and Nrf2 in SeC-treated cells. WiDr cells exposed with 50 μM of SeC for 24 hr, and then collected cell lysate. 100 μg of cell lysate were subjected to immunoprecipitation assay with 1 μg of anti-Keap1 antibody, followed by immunoblotting of anti-ubiquitin antibody. In Nrf2 ubiquitination assay, WiDr cells were treated with SeC for 9 hr. 500 μg of cell lysate were subjected to immunoprecipitation assay with 1.3 μg of anti-Nrf2 antibody, followed by immunoblotting of anti-ubiquitin antibody. **C** Immunoblotting of phospho-GSK3β and total GSK3β in cells treated with SeC for 24 h. The experiment was performed with three repetitive treatments

**
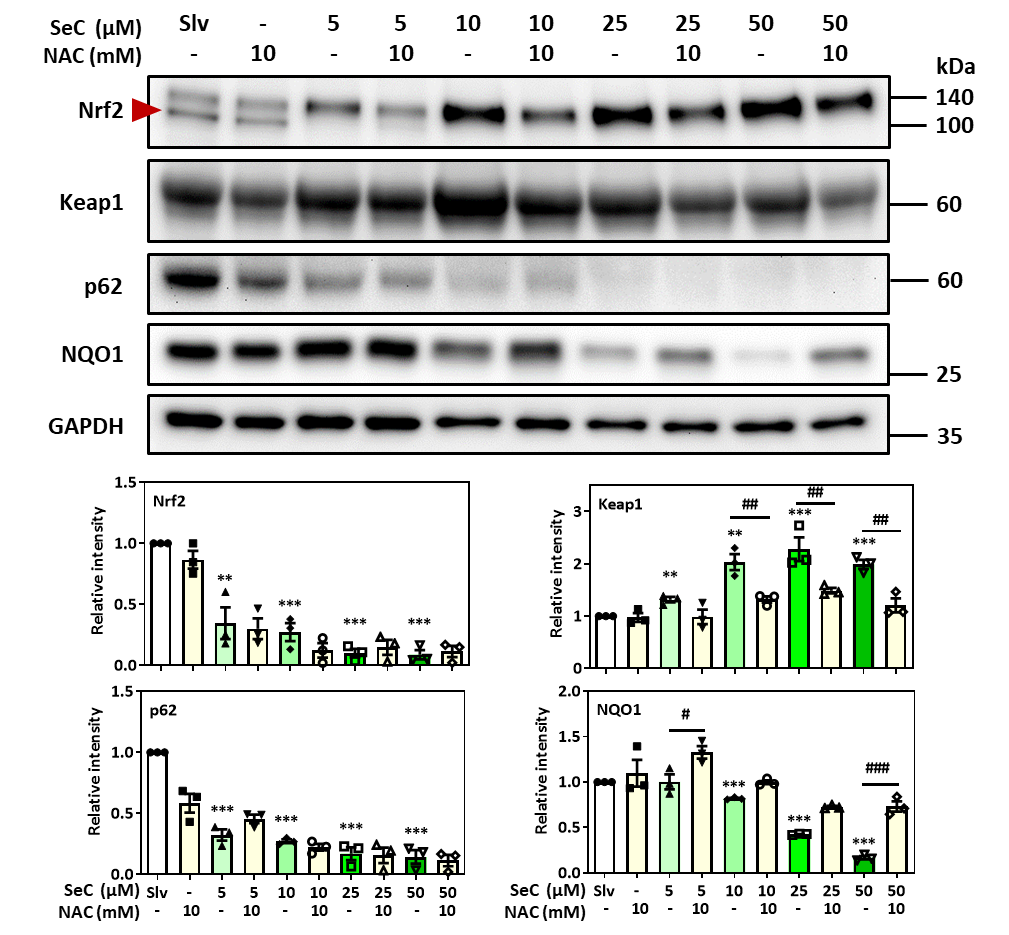
**

**Supplementary Fig. 5 Effects of NAC on the NRF2 pathway in SeC-treated WiDr cells after 24 h.**

Levels of phosphorylated NRF2 and NRF2 regulated proteins in SeC-treated WiDr cells after SeC treatment pretreated with or not pretreated with and NAC. GAPDH was used as loading control (n=3) (*P < 0.05, **P < 0.01, ***P < 0.001, compared with solvent control, ##P < 0.01, ###P < 0.001, compared with SeC-treated cells without antioxidant pretreatment, significance was determined by one-way ANOVA). The experiment was performed with three repetitive treatments.

**
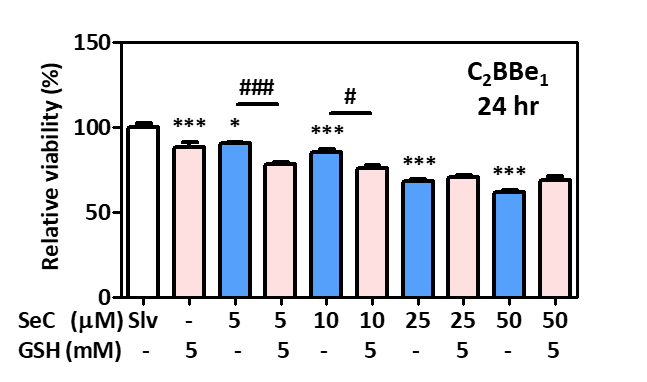
**

**Supplementary Fig. 6 Viability of SeC-treated C_2_BBe_1_ cells with or without GSH pretreatment after 24 h.** Relative viability of SeC-treated C2BBe1 cells with or without GSH pretreatment for 24 h. The experiment was performed with eight repetitive treatments. (n=8, *p < 0.05, **p < 0.01, ***p < 0.001, compared with solvent control; ##p < 0.01, ###p < 0.001, compared with SeC alone, significance was determined by one-way ANOVA).
