# Supplementary material for: Blockage of Nrf2 and autophagy by L-selenocystine induces selective death in Nrf2-addicted colorectal cancer cells through p62-Keap-1-Nrf2 axis
Source: Cell Death Dis. 2022 Dec 20;13(12):1060. doi: 10.1038/s41419-022-05512-2 (PMC9768144; doi:10.1038/s41419-022-05512-2)
Supplement: Supplementary file 2 — Supplementary material [file 41419_2022_5512_MOESM2_ESM.pptx]

## Slide 1
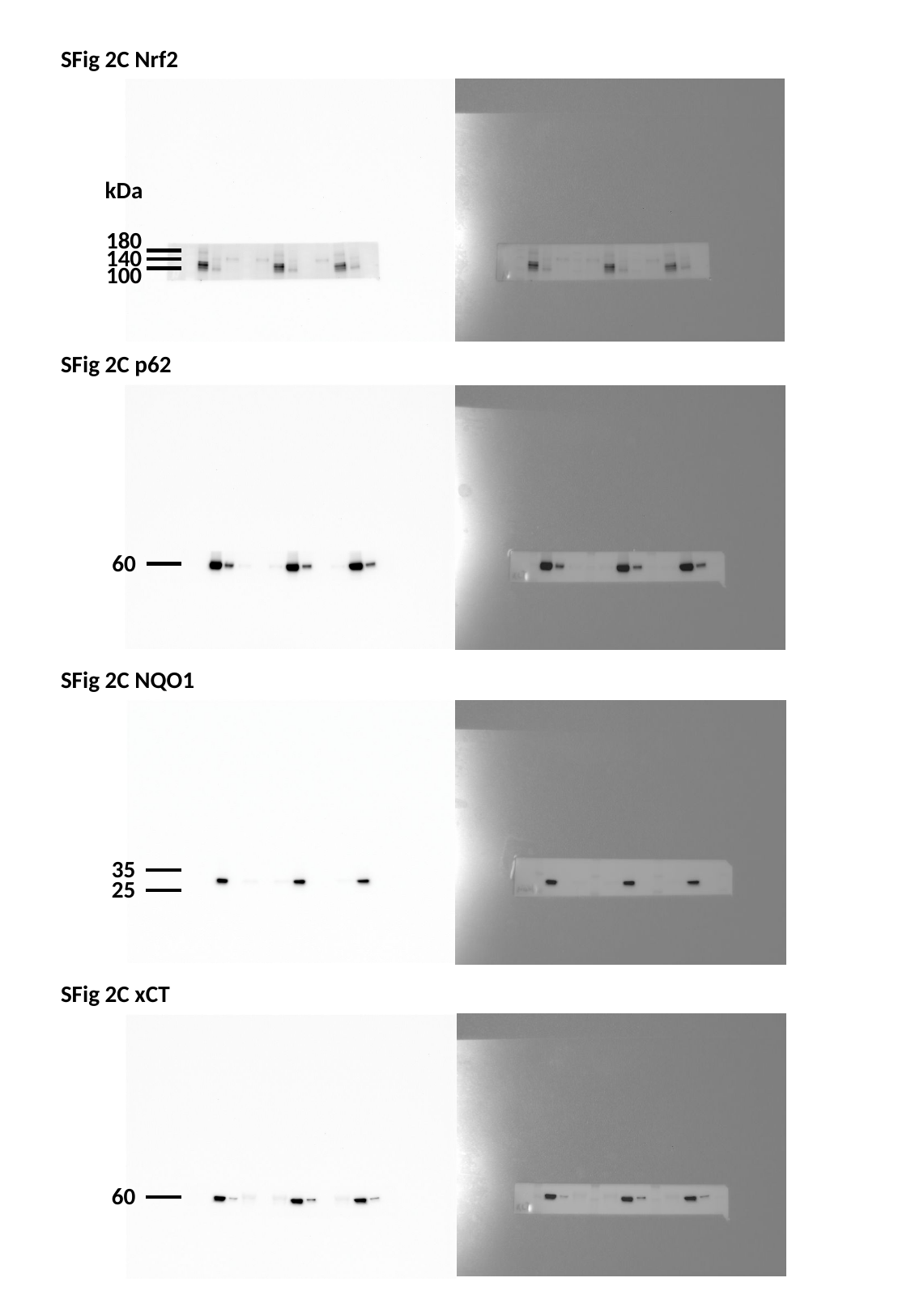

SFig 2C Nrf2
kDa
180
140
100
SFig 2C p62
60
SFig 2C NQO1
35
25
SFig 2C xCT
60

## Slide 2
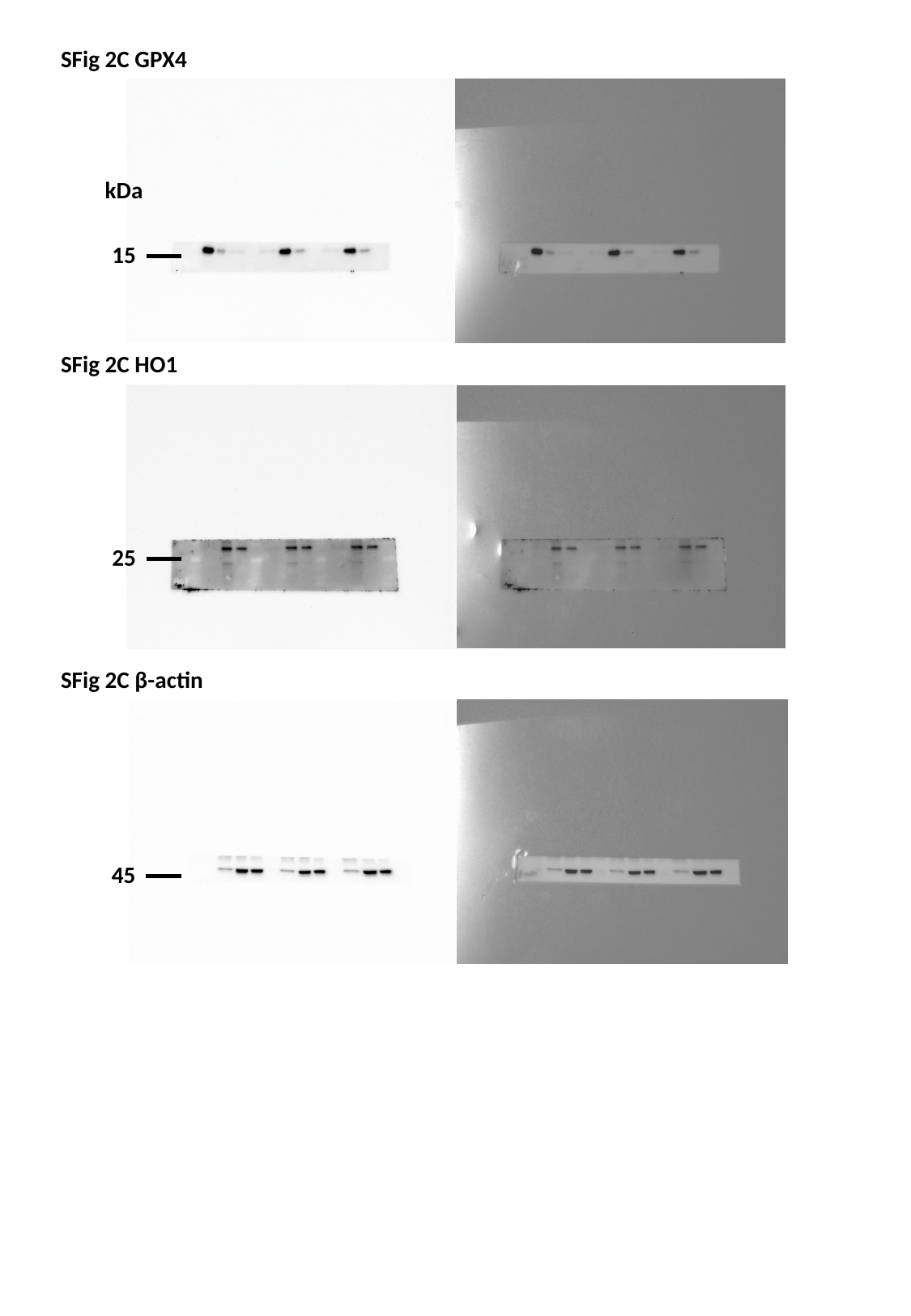

SFig 2C GPX4
kDa
15
SFig 2C HO1
25
SFig 2C β-actin
45

## Slide 3
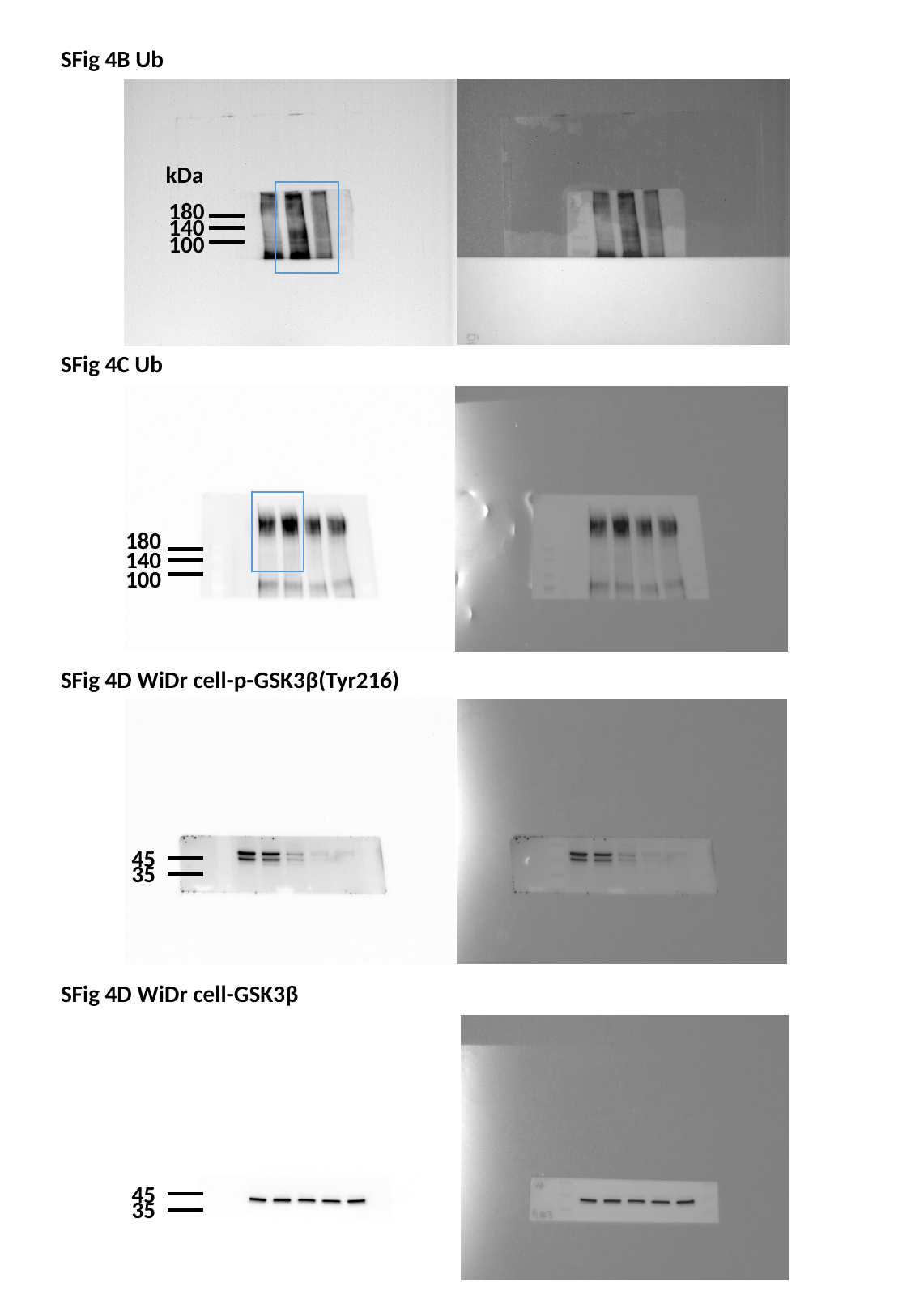

SFig 4B Ub
kDa
180
140
100
SFig 4C Ub
180
140
100
SFig 4D WiDr cell-p-GSK3β(Tyr216)
45
35
SFig 4D WiDr cell-GSK3β
45
35

## Slide 4
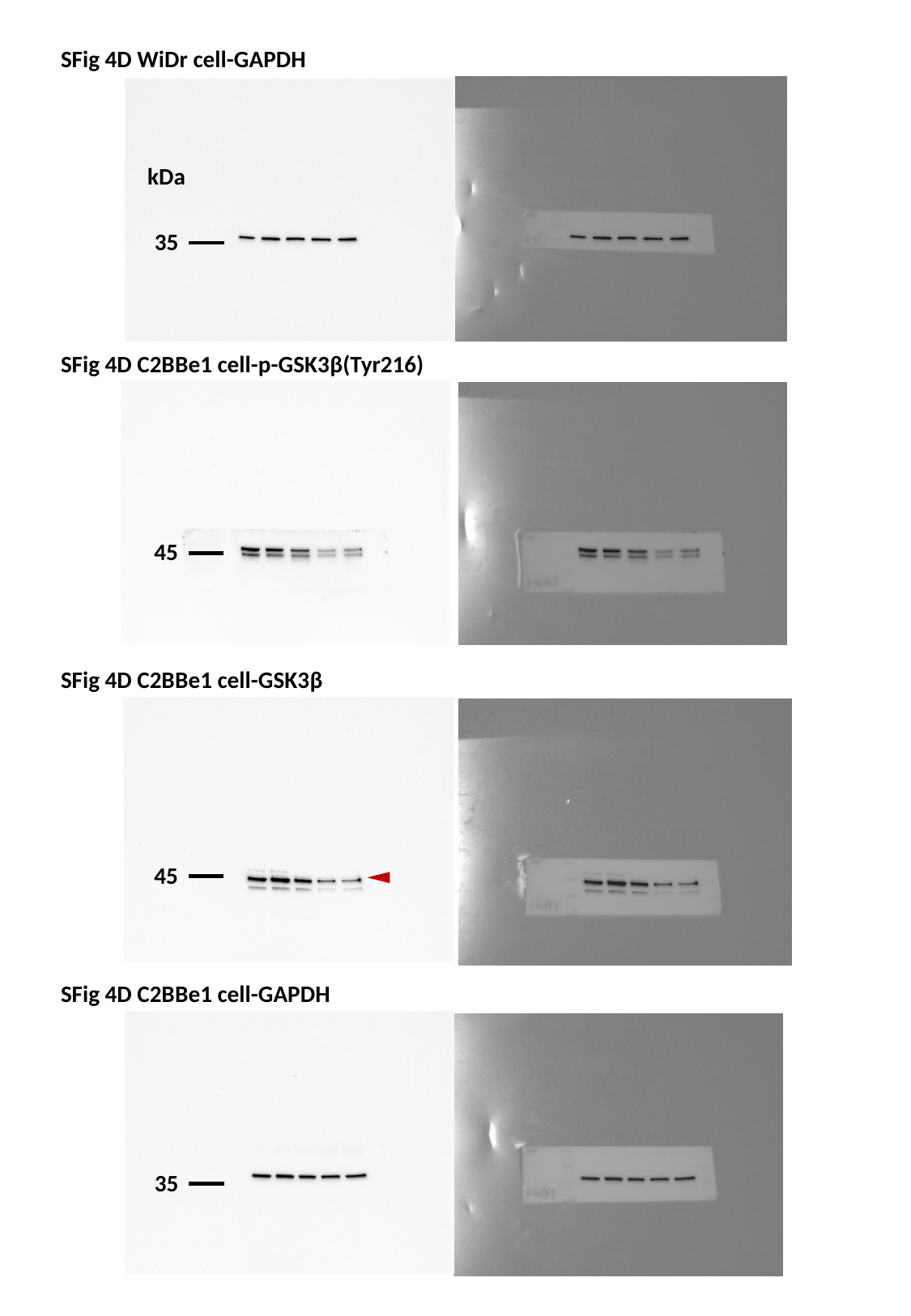

SFig 4D WiDr cell-GAPDH
kDa
35
SFig 4D C2BBe1 cell-p-GSK3β(Tyr216)
45
SFig 4D C2BBe1 cell-GSK3β
45
SFig 4D C2BBe1 cell-GAPDH
35

## Slide 5
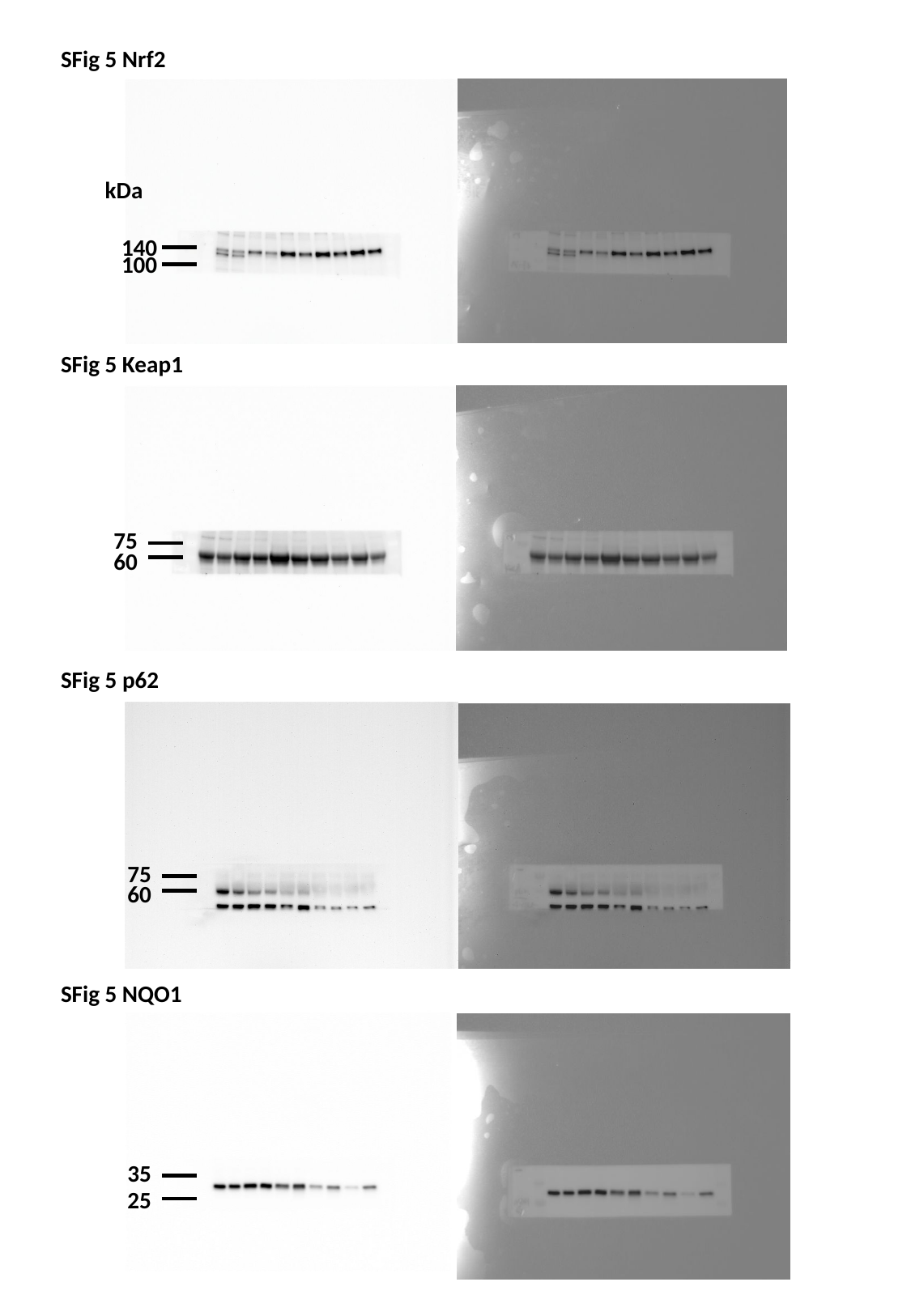

SFig 5 Nrf2
kDa
140
100
SFig 5 Keap1
75
60
SFig 5 p62
75
60
SFig 5 NQO1
35
25

## Slide 6
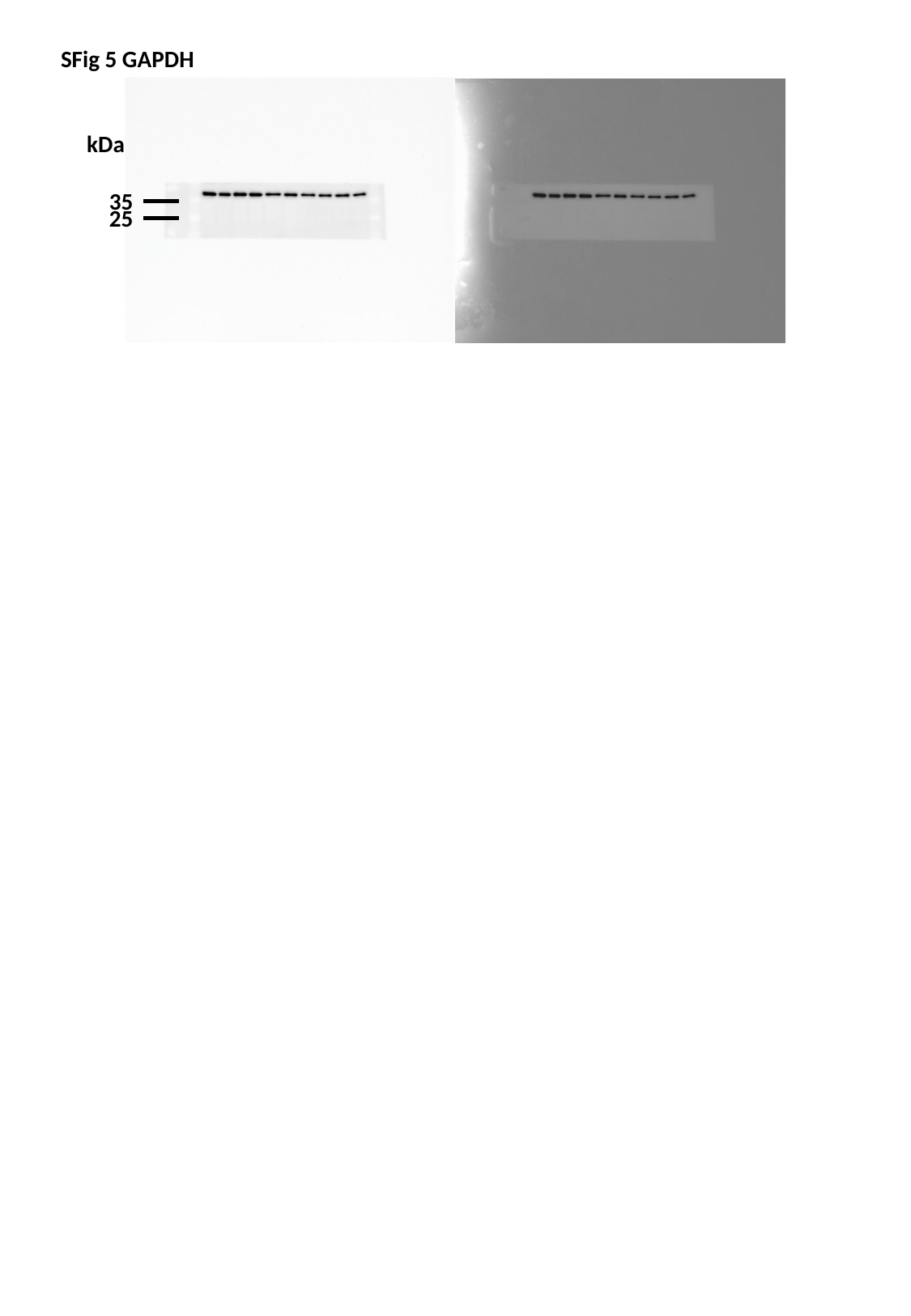

SFig 5 GAPDH
kDa
35
25
